# Conceptualizations of Cyberchondria and Relations to the Anxiety Spectrum: Systematic Review and Meta-analysis

**DOI:** 10.2196/27835

**Published:** 2021-11-18

**Authors:** Sandra K Schenkel, Stefanie M Jungmann, Maria Gropalis, Michael Witthöft

**Affiliations:** 1 Department of Clinical Psychology, Psychotherapy, and Experimental Psychopathology Johannes Gutenberg-University Mainz Mainz Germany

**Keywords:** cyberchondria, health anxiety, online health information seeking, anxiety, systematic review, meta-analysis

## Abstract

**Background:**

*Cyberchondria* describes the detrimental effects of health-related internet use. Current conceptualizations agree that cyberchondria is associated with anxiety-related pathologies and may best be conceptualized as a safety behavior; however, little is known about its exact underlying mechanisms.

**Objective:**

This systematic review and meta-analysis aims to give an overview of the conceptualizations of cyberchondria and its relation to anxiety-related pathologies, quantify the strength of association to health anxiety by using meta-analyses, highlight gaps in the literature, and outline a hypothetical integrative cognitive-behavioral model of cyberchondria based on the available empirical evidence.

**Methods:**

A systematic literature search was conducted using PubMed, Web of Science, and PsycINFO electronic databases. A total of 25 studies were included for qualitative synthesis and 7 studies, comprising 3069 individuals, were included for quantitative synthesis. The meta-analysis revealed a strong association of cyberchondria (*r*=0.63) and its subfacets (*r*=0.24-0.66) with health anxiety.

**Results:**

The results indicate that cyberchondria is a distinct construct related to health anxiety, obsessive-compulsive symptoms, intolerance of uncertainty, and anxiety sensitivity. Further studies should distinguish between state and trait markers of anxiety-related pathologies and use experimental and naturalistic longitudinal designs to differentiate among risk factors, triggers, and consequences related to cyberchondria.

**Conclusions:**

Health-related internet use in the context of health anxiety is best conceptualized as health-related safety behavior maintained through intermittent reinforcement. Here, we present a corresponding integrative cognitive-behavioral model.

## Introduction

### Background

The internet allows anonymous access to a huge amount of specific information and opinions from nearly everywhere, at any time, and at relatively low costs [1,2]. It is increasingly being used to research health-related questions. Approximately 60% to 80% of internet users search the web for health-related information [3-5]. Of all internet search queries, 2% have medical content [6]. In 2013, 35% of surveyed American adults reported that they had started at least one web-based search session with the specific purpose of figuring out which medical condition they themselves or another person suffered from [7]. Arguably, a web-based search for perceived symptoms or feared illnesses can be helpful. Lemire et al [8] found that individuals feel empowered (ie, competent and in control) by conducting health-related internet use. In addition, health-related internet use seems to enable patients to take a more active role in patient-doctor–relationships [9]. It opens new opportunities for illness prevention and health care, such as developing statistical models based on individual queries that might be used as a warning system, for example, for cancer [10]. However, there is also evidence of detrimental effects, such as an increase in worries or (health) anxiety, and as a consequence, an increase in the use of health care resources [11,12] (eg, 46% of a general population sample stated that health information found on the web led them to think they needed an appointment with a medical professional [7]).

In this context, journalists have coined the term *cyberchondria* [4,13] from the words *cyber*, referring to internet use, and *hypochondriasis*, referring to pathological health anxiety (HA). It is the belief in or the fear of having a serious disease, often without a matching medical condition. However, the term *cyberchondria* itself does not say anything about causality, the nature of the relationship between these two constructs, or the relevance of cyberchondria for patients with pathological HA. The most common definition of cyberchondria provided by Starcevic and Berle [14] postulates a bidirectional relationship. Elevated HA triggers health-related internet use, which in turn leads to amplification and, in the long term, to the maintenance of HA. Others classify cyberchondria-related behavior as a form of reassurance seeking that initially leads to an immediate decrease of HA but maintains HA in the long term through negative reinforcement [15].

Associations between cyberchondria-specific behavior and anxiety-related pathologies other than HA have also been proposed. First, a connection to obsessive-compulsive symptoms has been hypothesized [16-19], such that individuals experiencing greater cyberchondria also experience greater obsessive-compulsive symptoms. The interruption of other activities because of health-related internet use appears to be common in both constructs. Second, uncertainty about the seriousness of bodily symptoms and their appraisal as dangerous were proposed to be triggers for health-related internet use [1,14]. Several studies found medium-sized positive correlations between cyberchondria and intolerance of uncertainty (Multimedia Appendix 1 [20-23]). Furthermore, intolerance of uncertainty has been shown to moderate the impact of health-related internet use on HA [20,24]. The results suggest that the desire to avoid uncertainty and negative reactions to uncertainty are strongly associated with the experience of negative affective states because of health-related internet use [21,25]. Positive correlations were also found between anxiety sensitivity (ie, the tendency to interpret anxiety-related symptoms as signs of impending danger) and cyberchondria (Multimedia Appendix 2 [21,22,25]). Furthermore, anxiety sensitivity predicts cyberchondria in addition to the contributions of intolerance of uncertainty [25].

Different conceptualizations and theories have been developed that focus on certain aspects, such as emotional or behavioral consequences, cognitions, or characteristics of the search process itself; however, no consensus has yet been reached. The fact that cyberchondria is a current topic is also made clear by the fact that in 2019, for example, a systematic narrative review [26] and a meta-analysis of cyberchondria and HA [27] were published. Brown et al [26] presented an integrative cognitive-behavioral therapy (CBT) model of health-related internet use that distinguishes between reassuring health-related internet use on the one hand and problematic as well as compulsive health-related internet use on the other hand, according to the emotional consequences of this behavior. McMullan et al [27] found a meta correlation between HA and cyberchondria of *r*=0.62 (*P*<.001; n=10 studies).

### Objective

This review aims to (1) give an overview of the current state of research regarding existing theoretical conceptualizations of cyberchondria and its relation to anxiety-related pathologies (ie, broader compared with the previous focus on HA), (2) quantify the strength of associations between cyberchondria and HA by using a meta-analysis according to the current data situation (ie, year 2020), (3) highlight gaps in the current literature, and (4) outline a hypothetical integrative cognitive-behavioral model of cyberchondria based on the available empirical evidence. This model follows a new approach by integrating existing results in the context of elevated HA and by waiving the artificial conceptual separation of health-related internet use according to the valence of its emotional effects.

## Methods

### Protocol and Search Strategy

A systematic literature search was conducted according to the PRISMA (Preferred Reporting Items for Systematic Reviews and Meta-Analyses) guidelines [28,29], using the computerized databases PubMed, Web of Science, and PsycINFO. They were searched three times, first in February 2016, second in July 2017, and third in February 2020, to include the latest research findings. A keyword search was performed using the following search terms and logics: *cyberchondria, cyberchondriasis, health-related internet use AND health anxiety, health-related internet use AND hypochondriasis, illness-related internet use AND health anxiety,* and *illness-related internet use AND hypochondriasis,* as well as the German translations of these terms. Searches were restricted to study titles and abstracts.

### Inclusion and Exclusion Criteria

Only a few eligibility and exclusion criteria were specified to make this search as inclusive as possible. Studies were included if they (1) examined health-related internet use in the context of HA, (2) were published research papers or accepted manuscripts, (3) were written in English or German, and (4) investigated a general population or a health-anxious sample. Articles were excluded if they (1) reported a case study or data exclusively from children or adolescents or both, (2) were literature reviews or comments that did not postulate their own conceptualization or definition of cyberchondria, or (3) contained special characteristics that impeded the generalization of results (eg, investigation of a sample with symptoms outside the anxiety-related spectrum). All included research articles were already published in peer-reviewed journals.

### Data Extraction and Synthesis Process

**Figure 1:**
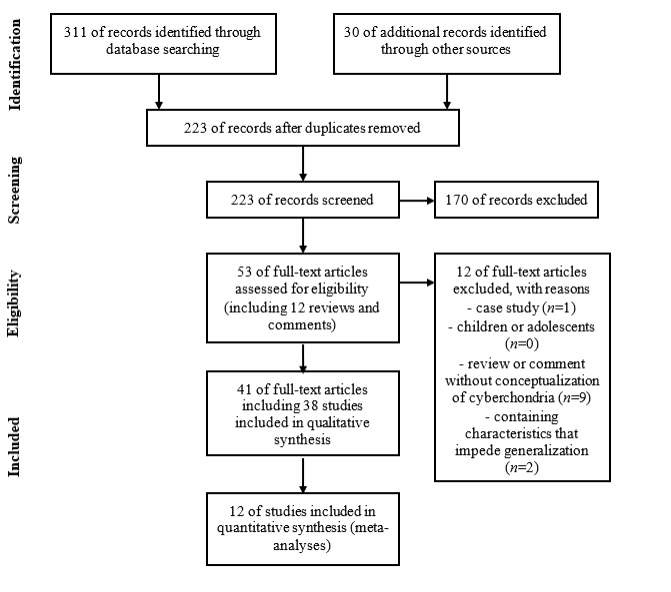
PRISMA (Preferred Reporting Items for Systematic Reviews and Meta-analyses) flow diagram of studies.

First, study titles were screened. Then, the abstract, method, and discussion sections were read consecutively (especially to detect assumptions concerning the concept or function of cyberchondria), followed by reading full texts. Studies that did not fulfill all eligibility criteria or that fulfilled one or more exclusion criteria were eliminated. The electronic search was supplemented by reading the reference lists of the retrieved studies to identify more potential literature. Then, information from each eligible study was extracted and tabulated. Extracted data included names of the authors, publication year, journal, country of origin, type of study (primary vs secondary), proposed conceptualization of cyberchondria (yes or no), research question, study design, number of reported studies per paper, sample characteristics (age, range, type, and total sample size), methodology (operationalization of the construct, type of hypothesis, and analysis), and main findings. No paper was excluded because of insufficient information being reported. Applying these criteria to the searches resulted in the inclusion of 38 studies (Figure 1). All studies proposing a conceptualization of cyberchondria were then picked (irrespective of whether the study was primary or secondary). Different elements of conceptualization (eg, suggestion of detrimental effects of health-related internet use) were then extracted, which, in turn, were used as categories to group the corresponding primary studies (eg, studies investigating the effects of health-related internet use). Accordingly, one primary study could be assigned to more than one group. All primary studies were assigned. In the following sections, we present the studies according to the assigned groups (ie, *Conceptualizations of Cyberchondria*, *Cyberchondria and Anxiety-Related Pathologies*, *Triggers for Health-Related Internet Use*, and *Consequences of Health-Related Internet Use*).

## Results

### Conceptualizations of Cyberchondria

Conceptualizations of cyberchondria differ in the proposed immediate impact of health-related internet use on HA, and thus, also regarding the function of cyberchondria-related behavior in the context of HA (ie, *cyberchondria as an Amplifier of HA* vs *Cyberchondria as a Safety Behavior*). Multimedia Appendix 3 [6,14,15,26,30,31] summarizes the current conceptualizations.

#### Cyberchondria as an Amplifier of HA

##### Cyberchondria as a Failed Safety Behavior

Starcevic and Berle [14] presented a definition of cyberchondria as “an excessive or repeated search for health-related information on the internet, driven by distress or anxiety about health, which only amplifies such distress or anxiety”; moreover, “it does not denote a diagnosis and occurs as part of health anxiety and hypochondriasis.” While giving a theoretical and empirically based framework, this definition also reformed the operationalization of cyberchondria by defining a process that includes a causal bidirectional relationship between cyberchondria and HA. It is important to underline that defining the precursors (HA as a trigger) and consequences (exacerbation of HA) of health-related internet use differentiates cyberchondria from merely searching the web for health-related information. Moreover, the multidimensionality of the construct was implied (eg, distress, excessiveness, and compulsion) [17]. Starcevic and Berle [14] hypothesized that cyberchondria-related behavior may be a form of reassurance seeking but that it should not be classified as a *classic* safety behavior, as in the short term, it increases anxiety instead of decreasing it [32]. If this definition is correct, it is important to explain how health-related internet use increases HA and why this behavior does not stop despite its adverse effects. In this regard, factors that amplify anxiety during health-related internet use are presented [33], which are assumed to set vicious circles in motion. They include the misinterpretation of the rank order of search results as pointing to the likely cause of a searched symptom [6,34] and the questionable trustworthiness of web-based health information [35]. However, current and partly inhomogeneous results raise doubts in this context [13,34,36].

##### Query Escalation

White and Horvitz [6] hypothesized that a process referred to as *query escalation* is highly relevant for increased HA during medical web-based searches. They defined cyberchondria as “the unfounded escalation of concerns about common symptomatology, based on the review of search results and literature on the Web.” Escalations, in turn, were defined as special cases of cyberchondria during health-related internet use. They were operationalized as a shift of attention concerning the content of the information sought: away from a probable cause (eg, dehydration) for a common symptom (eg, a headache) to a serious illness (eg, cancer), which is a very unlikely cause for the bodily sensation.

To investigate this process, 11,158 naturalistic logs of a large (N=515) nonclinical sample’s internet searches were analyzed [6]. Approximately 2% of all queries had medical content, and 5.3% of them escalated, thus verifying the existence of query escalations. However, as the direct effects on HA were not assessed, it could not be shown that this was a mediating process for experiencing increased anxiety. Evidence was found that searches were performed repeatedly, as 78.3% of all medical queries contained a symptom that was sought again within 2 weeks after the initial search. Moreover, the results indicated that health-related internet use occurred impulsively with a switch between longer abstinent episodes and episodes of intensive searching behavior, which might be indicative of an intermittent reinforcement pattern.

Singh and Brown [37] investigated the occurrence and consequences of query escalations in dependence on the HA level of their participants in a laboratory experiment. They found that escalations resulted in higher anxiety compared with nonescalations, regardless of previously existing HA, and highly health-anxious individuals were significantly more likely to escalate. Approximately 60% of all participants performed at least one escalated health-related internet use.

There are initial indications for the relevance of *query escalation* as a moderating mechanism for the negative effects of health-related internet use and these lead to the conclusion that attentional aspects might be of relevance in cyberchondria. In a consecutive study, White and Horvitz [34] showed that potentially alarming content (eg, *heart attack* and *medical emergency*) in captions, snippets, and URLs of search results influences the search-result click-through behavior so that this attribute makes them more likely to be selected.

##### Dimensional Conceptualizations of Cyberchondria

Cyberchondria was usually assessed using single items referring to the frequency or duration as well as the emotional consequences of health-related internet use [4,13,20]. These operationalizations neither took into account the postulated process character nor the multidimensionality of this construct [6,14]. In 2014, McElroy and Shevlin [17] developed the Cyberchondria Severity Scale (CSS), designed as a continuous measure of distress because of health-related internet use (according to previous conceptualizations [4,6,13,14]). This self-assessment questionnaire comprises 33 items that ask questions on a 5-point Likert scale about the frequency of web-based health-related behaviors. In contrast to previous assessment strategies, the CSS consists of five interpretable factors that reflect different dimensions of cyberchondria [16,17,38,39]. The factor *compulsion* reflects the different ways in which a web-based health-related search can unintentionally interrupt other web-based and offline activities. The dimension *distress* mirrors subjective negative emotional states because of health searches on the internet (stress, worry, anxiety, panic, and irritation). The factor *excessiveness* captures repeated searches for the same topic and the use of numerous sources. These 3 dimensions were also proposed by Starcevic and Berle [14]. Two additional subfacets are presented: all items loading on the factor *reassurance seeking* (hereafter called *reassurance*) indicate the felt need for reassurance from medical professionals triggered by information found on the web, representing a behavioral consequence of cyberchondria. The factor *mistrust of medical professionals* (hereafter called *mistrust*) reflects the conflict that web searchers have concerning whether to trust their medical professional over their own research results and self-diagnosis.

As detailed in Multimedia Appendix 4 [16-19,26,40,41] all CSS subscales showed moderate to strong intercorrelations, except for the *mistrust* factor, where only small or nonexistent associations were observed.

Although factor analyses revealed a five-factor structure of the CSS items, further analyses and observed correlation patterns raised doubts concerning the affiliation of *mistrust* to the concept of cyberchondria [16,38,39]. Higher-order factor analyses showed that a model consisting of a separate *mistrust* factor and a bifactor model comprising a general cyberchondria factor and 4 independent dimensions (*compulsion*, *distress*, *excessiveness*, and *reassurance*) best fit the data [16,38]. This showed that models generating a general cyberchondria factor had a superior fit to the data when *mistrust* was excluded. The four-factor structure was also found in a network analysis [42] that additionally revealed that no symptom seemed to be more central to the cyberchondria construct than others. On the other hand, Norr et al [38] pointed out that the findings regarding the *mistrust* factor may be attributable to method variance as all items of the scale are designed inversely. However, this approach has not been further investigated. Furthermore, qualitative data suggest that a certain mistrust of medical professionals and, specifically, of doctors, may play an important role [1]. In this context, a distinction has to be made between the affiliation of *mistrust* to the CSS and the affiliation of mistrust of doctors to cyberchondria; however, this question cannot be answered at this time. Further investigation is needed that takes into account the possibility that the CSS may not assess the construct in its entirety and that the general cyberchondria factor may represent a dimension of cyberchondria that is only weakly associated with *mistrust*. The original and revised CSS total scores (removing the *mistrust* items) were highly correlated (*r*=0.99 [16]).

Regarding convergent validity, the most important indicator for the CSS is its potential association with HA (see section *Health Anxiety*). Correlation analyses demonstrated that the CSS data were more strongly associated with *anxiety* (*r*=0.43; *P*≤.01) than with *stress* (*r*=0.37; *P*≤.01) and *depression* (*r*=0.24; *P*≤.01 [17]) using the short version of the Depression, Anxiety and Stress Scale [43]. The degree of association varies according to the theoretical similarities of the different constructs. Moreover, Fergus [16] showed that the CSS total score and the total score revised were more strongly associated with HA (*r*=0.59 and *r*=0.58, respectively; both *P*<.01) than with obsessive-compulsive symptoms (for both total scores, *r*=0.49; *P*<.01), as assessed using the Dimensional Obsessive-Compulsive Scale (DOCS) [44]. Barke et al [39] found medium positive correlations between the CSS global score and somatic symptoms (*r*=0.40; *P*<.01; assessed with the somatic symptom scale of the Patient Health Questionnaire [45,46]), depressive symptoms (*r*=0.31; *P*<.01; assessed with the short form of the Center for Epidemiologic Studies Depression Scale [47,48]), and health care use (*r*=0.29; *P*<.01; assessed with the Health Care Utilization questionnaire [49]).

As can be seen in Multimedia Appendix 4, the CSS subscales show different internal consistencies ranging from a high level for *compulsion* and *distress* to a good level for *excessiveness*, *reassurance*, and *mistrust*. The CSS total scale showed excellent internal consistency and split-half reliability without exception (α=.93-.96 [16,17,19,38,39]).

The first results show that the CSS seems to be unrelated to age [39], whereas results regarding sex differences are heterogeneous [2,39]. Too little information is currently available to finally evaluate these factors.

Other versions of the CSS regarding language (German [39] and Polish [40]) and length (short versions in English [50] and German [39]) show psychometric qualities comparable with the original version.

Jokić-Begić et al [51] developed a short questionnaire (4 items) to assess cyberchondria as a safety behavior. Besides predictors and negative outcomes, positive consequences were also intended to be measured; however, analyses revealed an unclear factor structure.

##### Cyberchondria as a Form of Problematic Internet Use

Problematic internet use describes the excessive use of the internet [52] for purposes other than searching for medical information and an inability to control that use [53], therefore reflecting behaviors from the fields of compulsion and addiction. Fergus and Dolan [30] define cyberchondria as a form of problematic internet use that is intended to reduce negative emotions but actually leads to greater subjective distress. To investigate this assumption, they used the Compulsive Internet Use Scale [54] and operationalized cyberchondria as the impact of health-related internet use on HA assessed with a single item. Individuals experiencing increased HA because of health-related internet use reported significantly greater levels of problematic internet use (mean 25.35, SD 11.58) compared with individuals experiencing no impact on (mean 18.63, SD 10.56; Cohen *d*=0.61; *P*<.01) or a decrease in HA (mean 21.76, SD 10.43; Cohen *d*=0.33; *P*<.01). These group differences stayed robust even after controlling for the frequency of health-related internet use and negative affect. These findings were supported by Fergus and Spada [31], who found a strong association between cyberchondria (operationalized by the CSS) and problematic internet use (*r*=0.59; *P*<.001). In this study, problematic internet use was assessed using the Problematic Internet Use Questionnaire [55], which consists of 3 dimensions (obsession, neglect of other activities, and control disorder). In addition, multiple linear regression with N=337 participants showed the robustness of this relationship (step 2; β=.41; *P*<.001) by controlling for age, gender, physical health, negative affect, and HA, where HA was the only covariate that accounted for variance in cyberchondria scores. Besides, Starcevic et al [42] found a stronger relationship between cyberchondria and problematic internet use than with HA. However, their network analysis showed that cyberchondria and problematic internet use were related but distinct constructs.

Fergus and Spada [31] concluded that cyberchondria could be viewed as a specific form of problematic internet use, indicating that concepts of problematic internet use may be of relevance to understanding cyberchondria. In addition, cognitive-behavioral treatments for problematic internet use may also be used to treat cyberchondria [30].

Moreover, Singh and Brown [11] found significant positive correlations between HA and 6 indicators of addiction to health-related internet use (inter alia, unsuccessfully trying to cut back, negative feelings regarding a real or anticipated loss, negative consequences, and increasing use over time).

Together, these findings underline the relevance of the compulsive and addictive aspects of cyberchondria-specific behavior.

##### Metacognitive Conceptualization of Cyberchondria

Fergus and Spada [22,31] hypothesized the conceptualization of cyberchondria according to the metacognitive model of emotional disorders [56]. Following this model, individuals engage in self-regulation strategies that maintain and worsen negative affective states because of their metacognitive beliefs. In terms of cyberchondria, this would mean that health-related images, memories, or thoughts trigger HA. At the same time, metacognitive beliefs are activated, which can be distinguished into two types: *positive* metacognitions associated with advantages of health-related worries (eg, “Considering all possibilities will help keep my mind at rest” [31]) and *negative* metacognitive beliefs associated with disadvantages or the uncontrollability of health-related worries (eg, “My thoughts are uncontrollable” [31]). To reduce the triggered HA, a self-regulation process, namely health-related internet use, is initiated. Plans for supposed successful self-regulation are represented as mentioned in metacognitive beliefs and additionally in beliefs about rituals and stop signals. Rituals refer to plans for coping with aversive inner emotional experiences, and stop signals refer to self-relevant goals that signal when to stop the self-regulation process. Negative metacognitive beliefs, especially regarding the performance of health-related internet use (eg, “Once I start searching I cannot stop” [31]), which can arise in the course, may lead to an increase in negative affect and, thus, lead to further self-regulation processes in the form of health-related internet uses, resulting in a repetitive and distress-evoking vicious circle.

The relevance of metacognitive aspects for cyberchondria was supported by several results from 3 web-based questionnaire surveys by the same authors [22,31], in which the Metacognitions Questionnaire–Health Anxiety (MCQ-HA; [57]) was used. It incorporates three metacognitive beliefs: thoughts can cause illness (MCQ-HA-C), biased thinking (MCQ-HA-B; eg, “Worrying about my health will help me cope”), and thoughts are uncontrollable (MCQ-HA-U). In all studies, zero-order correlation analyses revealed that all metacognition subscales were significantly correlated with cyberchondria (MCQ-HA-C: *r*s=0.32-0.49, all *P*<.001; MCQ-HA-B: *r*s=0.47-0.58, all *P*<.001; and MCQ-HA-U: *r*s=0.51-0.66, all *P*<.001).

Hierarchical multiple linear regression analyses statistically controlling for relevant covariates (ie, age, gender, physical health status, HA negative affect, and anxiety sensitivity) showed that *negative* metacognitions (ie, uncontrollability of thoughts [22,31], beliefs about rituals [22], and beliefs about stop signals [22]) and obsessive-compulsive symptoms [22] contributed significantly to the variance in cyberchondria. However, the analyses yielded inhomogeneous findings regarding the relevance of *positive* metacognitive beliefs [22,31]. Interestingly, neither anxiety sensitivity nor intolerance of uncertainty shared significant, unique associations with cyberchondria [22]. A supplemental analysis using HA as the criterion variable (instead of cyberchondria) and cyberchondria as a covariate revealed that beliefs about rituals and stop signals distinguish cyberchondria from HA.

In summary, the presented results support the importance of metacognitive aspects in the conceptualization of cyberchondria. Nevertheless, the proposed function as a predisposing and maintaining factor in the cyberchondria process needs to be further investigated. In particular, the heterogeneous findings regarding the relevance of *negative* (vs *positive*) metacognitive beliefs need further attention to clarify if they have a unique function in maintaining health-related internet use despite its adverse effects.

#### Cyberchondria as a Safety Behavior

The cognitive-behavioral model of HA and hypochondriasis by Warwick and Salkovskis [58] postulates that the perception of normal bodily symptoms and their misinterpretation as harmful and as a sign of a serious illness as well as the mechanism of somatosensory amplification [59] lead to negative affect (eg, anxiety and worry). Safety behavior is exhibited to reduce the negative affective states. Bleichhardt and Weck [15] classified health-related internet use as a new form of reassurance-seeking behavior that is carried out on the internet rather than by reading books or magazines or visiting a physician.

Support for this categorization of cyberchondria was given by Newby and McElroy [60]. They adapted regular strategies for reducing safety behavior to cyberchondria and integrated them into an internet-delivered CBT for HA. Strategies comprised, for instance, increased awareness of the frequency and personal cost of health-related internet use, activity scheduling, and reduction of excessive health-related internet uses through a behavioral experiment. Analysis of variance analyses revealed a significant reduction in cyberchondria pre- and post-CBT (within-group Hedges g*=*1.57, 95% CI 1.05-2.09). This reduction was greater than that in an active control group (Hedges g=1.1, 95% CI 0.60-1.61; time by group, *F*_1,67_=25.41; *P*<.001). Mediation analyses showed that decreases in HA were partly mediated by a reduction in cyberchondria. Nevertheless, these promising results have to be interpreted in the light of several limitations, such as the influence of other therapeutic strategies and the moderate correlative association between cyberchondria and HA. Changes in cyberchondria might be reflective of changes in HA rather than of a unique process.

In 2019, the first CBT model was presented by Brown et al [26] based on a comprehensive review of existing data accounting for reassurance-related and compulsive elements.

According to the model, health-related internet use is commonly performed to eliminate health threats and is moderated by metacognitive beliefs (eg, “I need HIU to control my anxiety”), among other factors. Depending on the results of the health-related internet use, two possible forms of problematic health-related internet use can occur: first, if the search results can eliminate the perceived threat, relief is experienced, and health-related internet use is negatively reinforced. Consequently, health-related internet use is terminated, and metacognitive beliefs regarding the beneficial effects of this strategy are developed. This form is termed *pathological health-related internet use* and describes health-related internet use functioning as a reassurance-seeking behavior in the context of HA (ie, cyberchondria, although the authors recommend not to use this term because of ambiguities in the definition). A second possible outcome occurs if search results strengthen the perceived threat. Resulting anxiety and worry lead to the continuation of health-related internet use, even to the point of query escalation. In consequence, metacognitive beliefs about the internet search itself and its possible negative consequences (eg, “I can’t control my HIU”) are developed and lead to distress. This form is termed *compulsive health-related internet use*. The perceived threat does not focus on health (in contrast to pathological health-related internet use) but on the internet search itself. Individuals affected then might feel stuck and out of control regarding health-related internet use.

### Cyberchondria and Anxiety-Related Pathologies

In this section, show the associations between several anxiety-related pathologies (ie, HA, obsessive-compulsive symptoms, intolerance of uncertainty, anxiety sensitivity, and pain catastrophizing) and health-related internet use. Moreover, we discuss the distinctiveness of cyberchondria to anxiety-related pathologies.

#### Health Anxiety

All the aforementioned concepts and theories contain interrelations between HA and cyberchondria. For instance, Starcevic and Berle [14], as well as Brown et al [26], postulated that individuals who previously suffered from elevated HA are especially likely to experience negative affective states because of health-related internet use. Therefore, the following section illuminates the results regarding the relationship between HA and cyberchondria and is subdivided by the existing operationalizations of cyberchondria (by the CSS, including our meta-analyses, and by frequency and duration). As HA is seen as a dimensional rather than a categorical construct [61,62], findings from its whole spectrum are relevant.

#### A Meta-analytic Integration of Cyberchondria Operationalized by the CSS

As cyberchondria is supposed to be a multidimensional construct, it is conceivable that its different dimensions have dissimilarly strong relations to HA [38]. Multimedia Appendix 5 [18,19,38-40,63,64] displays the corresponding correlation coefficients. Individual dimensions have differing but significant associations with HA, ranging from high correlations with *distress* and *excessiveness*, moderate to high correlations with *reassurance* and *compulsion,* to moderate correlations with *mistrust*. To quantify the associations between (1) HA as a trait and cyberchondria, as well as between (2) HA and the different dimensions of cyberchondria as operationalized by the CSS, we conducted several meta-analyses. This examination is of particular importance to test the validity of the cyberchondria concept, as postulated by Starcevic and Berle [14].

#### Description of Included Studies

For the described purpose, we examined studies included in this review that reported results regarding a correlational hypothesis between a standardized measure of HA and the CSS total scale and subscales. On the basis of the aforementioned results regarding the questionable affiliation of the *mistrust* factor with the cyberchondria construct (or at least with the CSS total scale), only results that did not include the 3 *mistrust* items were incorporated. We contacted the authors of the primary studies to obtain missing data (eg, coefficients regarding the CSS total scale or subscales and internal consistencies). They were included if available. Quality ratings were conducted for the included articles, whereby the quality criteria according to Brown and Reuber [65] were adjusted based on the characteristics of the available studies (eg, some criteria were also met by all studies included in this study, such as correlative design and standardized measuring instruments, which were then not used as criteria). To assess the quality of the studies and especially the generalizability of the results, the following criteria were used and evaluated (by the 2 authors SKS and SMJ): consecutive sampling; validity items or data check, for example, missing values; control of possibly confounding variables; sufficiently reported inclusion and exclusion criteria; sufficiently reported sample characteristics; and approximate representativeness of the sample. According to Brown and Reuber [65], these ratings were used to calculate the overall quality of the study methods, which was defined as the proportion of items given a *yes* rating in combination with a sufficiently large sample size. We rated the sample size according to Cohen [66], who suggested that 85 participants were needed to detect a medium effect size (*r*=0.3), given an α level of .05 and power of 0.8. The overall quality was rated as high (≥80% *yes*, equating to no more than one of the methodological standards given a *no* rating and N≥85), medium (50-79% *yes* and N≥85), low (20-49% *yes* and N≥85), and unacceptable (<20% *yes* or N<85). Any unacceptable studies were excluded from the meta-analyses. Interrater reliability (between the 2 authors SKS and SMJ) across all categories was κ=0.75, indicating a substantial interrater agreement [67]. Quality ratings are provided in Multimedia Appendix 6 [16,21,22,25,31,39,40,63,64]. Of the 12 studies rated, 8 (67%) were judged to be of high quality, 3 (25%) of medium quality, 1 (8%) of low quality, and no study was rated to be of unacceptable quality, resulting in 12 studies that were included in the first meta-analysis regarding the first question (Table 1). Owing to unavailable missing data, 17% (2/12) of these studies were not considered in the meta-analysis regarding the second question, resulting in 10 studies for the second question.

**Table 1:** Details of studies included in the meta-analyses to quantify the association between HA^a^ and cyberchondria operationalized by the CSS^b^.

First author, year	Effect size, *r*	N	Age, mean (SD)	Sex (female), n (%)	HA (Cronbach α)	HA total score, mean (SD)	CSS (Cronbach α)	Ex^c^: medical condition	In^d^: perfor-ming health-related internet use	Country
Fergus, 2014 [16]	0.59	539	31.3 (9.9)	234 (43.4)	SHAI^e^ (.91)	11.12 (6.91)	CSS (.96)	Yes	Yes	United States
Fergus, 2015 [21]	0.62	578	31.2 (9.8)	253 (43.7)	WI^f^ (.91)	13.27 (5.06)	CSS (.95)	Yes	No	United States
Norr, Albanese et al, 2015 [25]	0.53	526	34.9 (12.4)	364 (69.2)	SHAI (.9)	14.40 (7.86)	CSS (.95)	No	No	United States
Barke et al, 2016, study A [39]	0.605	499	29.1 (10.4)	367 (73.6)	mSHAI^g^ (.9)	—^h^	German CSS (.93)	No	No	Germany
Barke et al, 2016, study B [39]	0.581	292	24.2 (4.1)	223 (76.4)	mSHAI (.93)	—	German short form of the CSS (.83)	No	No	Germany
Fergus and Russell, 2016 [19]	0.51	375	31.6 (10.2)	177 (47.3)	MIHT^i^ (.9)	—	CSS (.95)	Yes	No	United States
Fergus and Spada, 2017, study 2 [31]	0.67	260	32.9 (9.2)	106 (40.8)	WI (.92)	13.66 (5.48)	CSS (.95)	No	Yes	United States
Fergus and Spada, 2018, study 1 [22]	0.56	330	19.4 (2.1)	220 (66.6)	WI-6 (.89)	—	CSS (.95)	No	Yes	United States
Fergus and Spada, 2018, Study 2 [22]	0.61	331	38.7 (10.4)	177 (53.5)	WI-6 (.92)	—	CSS-15–Revised (.88)	No	Yes	United States
Bajcar et al, 2019^j^ [40]	0.56	240	26.5 (11.1)	203 (57.1)	SHAI (.93)	—	CSS-PL (.95)	No	No	Poland
Gibler et al, 2019^j^ [63]	0.58	221	19.2 (1.7)	156 (70.6)	SHAI (.89)	10.86 (6.12)	CSS (.95)	No	No	United States
Mathes et al, 2019 [64]	0.61	462	36.56 (12.9)	297 (64.3)	SHAI (.92)	TI^k^: 7.18 (4.41); FOI^l^: 3.13 (3.00)	CSS (.96)	No	No	United States

^a^HA: health anxiety.

^b^CSS: Cyberchondria Severity Scale.

^c^Ex: exclusion criterion.

^d^In: inclusion criterion.

^e^SHAI: Short Health Anxiety Inventory.

^f^WI: Whiteley Index.

^g^mSHAI: modified version of the Short Health Anxiety Inventory.

^h^Missing data.

^i^MIHT: multidimensional inventory of hypochondriacal traits.

^j^Excluded from the second meta-analysis (association between dimensions of cyberchondria and HA) because of unavailable data.

^k^TI: subscale thought intrusion of the SHAI.

^l^FOI: subscale fear of illness in SHAI.

#### Study Characteristics

Most studies were conducted in the United States (9/10, 90%). HA was mostly operationalized by the Short Health Anxiety Inventory (SHAI [68]) or a short version (7/10, 70%). Approximately 40% (4/10) used the Whiteley Index [41], and 10% (1/10) used the multidimensional inventory of hypochondriacal traits [69].

#### Participant Characteristics

In total, 4653 participants were included in the meta-analyses, with a mean age of 30.5 years; 58.3% were women. Approximately 30% (3/10) of studies excluded participants who reported that they suffered from a diagnosed medical condition, and 40% (4/10) of studies only included participants who stated that they regularly performed health-related internet use.

#### Data Analysis

**Figure 2:**
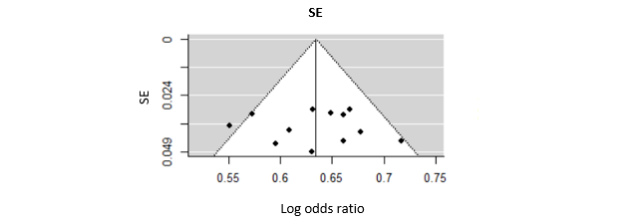
Funnel plot of integrated studies.

The random-effects model, considered to be the most appropriate in applied sciences was chosen [70,71]. This model is based on the assumption that the population effect differs randomly from study to study and accounts for within- and between-study variability [72,73]; therefore, more general inferences can be made compared with fixed-effect models. For statistical evaluation, the method provided by Hunter and Schmidt [74,75] was applied using the *metafor* software package in R (R Foundation) [76], following Viechtbauer’s [77] recommendations. This method aims to investigate relations on the level of constructs rather than measured values; therefore, it allows correction for the effects of several statistical artifacts, inter alia, concerning measuring accuracy (or more specifically for internal consistencies [78]). Potential publication bias was explored using a funnel plot (Figure 2). The Egger regression asymmetry test was used to detect publication bias. These analyses were also performed using the *metafor* software package in R [76]. For all the results, a two-sided *P* value of ≤0.05 was considered significant.

#### Meta-analytic Results

**Figure 3:**
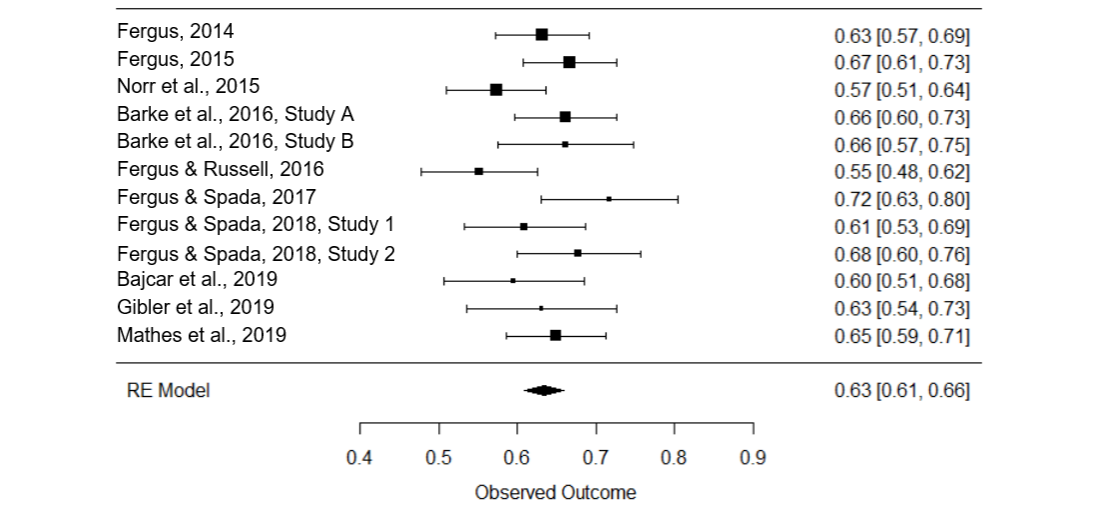
Forest plot of integrated study effects and meta-effect regarding the association between cyberchondria and health anxiety.

First, correlation coefficients were corrected for measurement error (Cronbach α [74]) of the instruments used to assess HA and cyberchondria. To quantify the association between these two constructs (question 1), a model with no moderators was estimated, revealing a small heterogeneity of integrated effects (I^2^=27.8%; *Q*_11_=16.7; *P*=.12 [79]); so no moderator analysis was conducted. A strong positive association between HA and cyberchondria was found (*r*=0.63; *P*<.001, 80% CI 0.61-0.66). Figure 3 shows the integrated effects. The Egger regression analysis showed that publication bias was not present (z=0.25; *P*=.80). Another meta-analysis concerning the strong positive association between HA and cyberchondria closely corresponded to our findings (*r*=0.62; *P*<.001 [27]).

**Figure 4:**
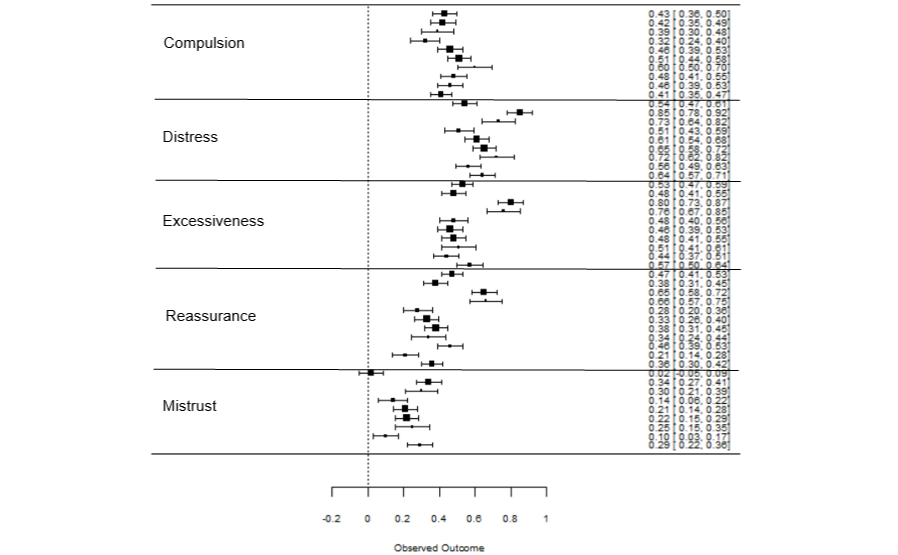
Forest plot of integrated study effects regarding the association between cyberchondria dimensions and health anxiety.

To quantify the association between the different dimensions of cyberchondria and HA (question 2), a second model was estimated that integrated the corrected correlation coefficients between HA and all subscales of the CSS (Figure 4).

In line with our expectations, high heterogeneity of effects was found (I^2^=95.59%; *Q*_44_=1020.81; *P*<.001). Therefore, subscales were included in the model as moderators and accounted for R^2^=67.26% of the variance. Table 2 shows the results of this meta-regression analysis. All subscales showed significant, small to strong associations with HA, and most strongly with *distress* and *excessiveness*.

**Table 2:** Results of meta-regression to quantify the association between cyberchondria dimensions and health anxiety.

Dimension of cyberchondria	β	*P* value	95% CI
Compulsion	.46	<.001	0.23-0.69
Distress	.66	<.001	0.43-0.89
Excessiveness	.59	<.001	0.36-0.83
Reassurance	.44	<.001	0.21-0.67
Mistrust	.24	<.05	0.01-0.47

##### Correlational Results Regarding Subfacets of HA

Correlation analyses further revealed that cyberchondria clusters most strongly correlated with the affective component of HA, more precisely with health worries (*r*=0.57; *P*<.01 [19]; Multimedia Appendix 5). This finding is consistent with the results of Norr et al [38], who found relations between cyberchondria and the SHAI, which is argued to primarily assess an affective component of HA [24]. As worry was found to be linked to safety behavior [80], the authors suggest that health-related internet use may be an activity performed to reduce health worries.

##### Cyberchondria Operationalized by Frequency and Duration of Health-Related Internet Use

Until the publication of the CSS [17], cyberchondria was assessed in two parts using two items: (1) frequency or duration of health-related internet use, and (2) its effects. Corresponding studies are detailed in Multimedia Appendix 7 [4,11,13,20,36,81,82]. Mostly moderate positive correlations were reported, indicating that the more health-anxious a person was, the more frequently and longer he or she searched the web for health- and illness-related information. This association was also found when HA was not operationalized continuously but dichotomously by ranking the SHAI scores of participants and using the bottom and top quartiles [4]. TePoel et al [81] observed a moderate positive association between HA and health-related internet use, indicating that individuals with higher HA reported more health-related internet use. This was true for both HA groups, clinical (β=.26; *P*<.001) and nonclinical (β=.29; *P*<.001).

##### Obsessive-Compulsive Symptoms

It is hypothesized that cyberchondria is connected with obsessive-compulsive symptoms. Correlational studies have used DOCS [44] to assess these. It consists of 4 dimensions: *contamination*, *responsibility for harm* (hereafter called *responsibility*), *unacceptable thoughts* (hereafter called *thoughts*), and *symmetry*. Each is judged for time spent, avoidance, distress, interference, and attempts to control. It was shown that the DOCS total scale is significantly correlated to the CSS total scales with (*r*=0.49, *P*<.01 [16]; *r*=0.38, *P*<.01 [40]) and without *mistrust* (*r*=0.49, *P*<.01 [16]; *r*=0.56, *P*<.001 [22]; *r*=0.38, *P*<.0 [40]) and to all CSS subscales (*distress: r*=0.50, *P*<.01 [16]; *r*=0.43, *P*<.01 [40]; *compulsion: r*=0.46, *P*<.01 [16]; *r*=0.34, *P*<.01 [40]; *excessiveness: r*=0.35, *P*<.01 [16]; *r*=0.31, *P*<.01 [40]; *reassurance*: *r*=0.27, *P*<.01 [16]; *r*=0.23, *P*<.01 [40]; *mistrust: r*=0.11, *P*<.01 [16]; *r*=0.07, not significant [40]), such that individuals with greater obsessive-compulsive symptoms experience greater cyberchondria. Moreover, as detailed in Multimedia Appendix 8 [18,19,40]*,* all CSS and DOCS subscales showed significant and mostly moderate intercorrelations. No statement can be made about the *mistrust* factor as it was excluded from the primary analyses. Interestingly, correlation coefficients were consistently smaller in the study conducted by Fergus and Russell [16] compared with those by Norr et al [18]. This might originate from a range restriction because of stricter eligibility criteria used by Fergus and Russell [19] regarding the performance of health-related internet use and the nonexistence of a diagnosed medical condition.

Although an association between cyberchondria and obsessive-compulsive symptoms was confirmed, different hypotheses exist about the nature of this relationship. First, it is supposed that obsessive-compulsive symptoms are an essential part of the cyberchondria construct. As was seen in section *Dimensional Conceptualizations of Cyberchondria*, McElroy and Shevlin [17] argue that health-related internet use can interrupt other web-based and offline activities. The results regarding the CSS dimension *compulsion* are reported in the aforementioned section. Here, we recap the results briefly. The subscale correlates highly with the CSS total scale (*r*=0.82; *P*<.01) and shows moderate to strong correlations with the other CSS subscales (*r*=0.26-0.80, excluding *mistrust*). Factor analyses [16,38] showed that *compulsion* seems to be part of a higher-order cyberchondria factor.

Another group of authors hypothesized that obsessional and compulsive behavioral elements contribute to the maintenance of cyberchondria [26]. Starcevic and Berle [14] suggested that maintenance is because of the combination of (1) an *obsessional doubt* regarding the validity and sufficiency of information found on the web, and (2) a hope to find the one *perfect* explanation for the symptoms experienced, which may represent a compulsive element of the cyberchondria construct. Following this argument, the subject would assume that continuing to perform health-related internet use would increase the probability of finding the *ultimate* answer, and for this purpose, any anxiety triggered by health-related internet use is acceptable. However, no empirical findings exist to address this hypothesis. Norr et al [18] postulated maintenance of health-related internet use because of a positive bidirectional association between cyberchondria and symptoms related to the DOCS subscale *contamination*. If health-related internet use is carried out to obtain reassurance, this behavior may lead to an increased obsession with physical health, owing to the strong link between cyberchondria and HA. This, in turn, may lead to an increased urge to prevent contamination and illnesses, for example, by increased hand washing. This amplified obsession with health may lead to further health-related internet use. Moreover, it is conceivable that if individuals with cyberchondria believe that health-related internet use gives them the power to prevent future illnesses, there may be a connection to the DOCS subscale *responsibility*. The *symmetry* and *thoughts* subscales are postulated to be unrelated. The structural equation model confirmed the predicted unique relationships. This model, controlling for negative affect (Positive and Negative Affect Schedule [83]) and HA (SHAI [68]), provided a good fit to the data (χ²=2954.63; *P*<.05; CFI=0.94; Tucker–Lewis index=0.93; root mean square error of approximation=0.05; 90% CI 0.05-0.06). Norr et al [18] concluded that a co-occurrence of both constructs and repeated health-related internet use might function as a safety behavior to reduce contamination concerns or responsibility for harm.

Another approach by Fergus and Russell [19] hypothesized that the existing findings regarding associations are because of a redundancy of the constructs. Health-related internet use may represent the behavioral component of both HA (eg, reassurance seeking) and obsessive-compulsive symptoms (eg, neutralizing behavior). They conducted confirmatory factor analyses to examine whether intercorrelations among these 3 constructs were best represented by indicator loadings on the same or separate latent constructs. Analyses showed that cyberchondria seems to be a distinct construct from HA and obsessive-compulsive symptoms, although they are related as a correlated three-factor model provided the best fit to the data (χ²_(41)_=149.3; comparative fit index=0.96; nonnormed fit index=0.95; standardized root mean square residual=0.06; root mean square error of approximation=0.08; 90% CI 0.69-0.99). In this model, a latent correlation of *r*=0.58 (*P*<.001) between cyberchondria and obsessive-compulsive symptoms was observed. This finding of distinctness of both constructs was confirmed by a network analysis by Starcevic et al [42].

In summary, the available empirical evidence is insufficient to finally characterize the nature of the relationship between cyberchondria and obsessive-compulsive symptoms. Moreover, it remains unclear whether obsessive-compulsive symptoms contribute to the maintenance of cyberchondria or vice versa. One clear shortcoming of research to date is that exclusively cross-sectional questionnaire-based surveys exist, which do not allow the investigation of processes and causality. Experimental studies and longitudinal data are needed to answer these questions.

##### Intolerance of Uncertainty

Intolerance of uncertainty is defined as a dispositional fear of the unknown [84]. It captures the inability to tolerate the uncertainty of ambiguous situations. It was found to comprise 2 dimensions, which are operationalized in the multidimensional Intolerance of Uncertainty Scale [85]: *prospective* intolerance of uncertainty reflects perceptions of threat as well as implications of future uncertainty. *Inhibitory* intolerance of uncertainty mirrors the desire to avoid uncertainty and behavioral symptoms of apprehension when faced with it. Considering intolerance of uncertainty as a risk factor for cyberchondria seems to be justified because of its substantial associations with HA [25,86].

The results from a qualitative interview study by Singh et al [1] support the relevance of uncertainty about whether one has an undiagnosed health issue for developing a felt need to go on the web and search for medical content. An association was confirmed by mainly medium-sized correlations (Multimedia Appendix 1), and a network analysis also revealed that both constructs were distinct [42]. In particular, *inhibitory* intolerance of uncertainty may be a risk factor for experiencing distress because of health-related internet use [21,25].

In addition, during health-related internet use, multiple medical possibilities are presented to the user [6], and for people with high intolerance of uncertainty, ambiguous situations were found to be distressing. Moreover, creating catastrophic interpretations of unclear health information is only related to HA at a high intolerance of uncertainty [24]. The moderating role of intolerance to uncertainty on the relationship between cyberchondria and HA seems conceivable to explain why some individuals experience worse HA as a consequence of health-related internet use, whereas others do not (see section *Consequences of Health-Related Internet Use*). A regression analysis by Fergus [20] confirmed this finding. The relationship between the frequency of health-related internet use and HA because of these searches was significant at high levels of intolerance of uncertainty (b=0.27; partial *r*=0.21*; P*<.01) but not at low (b=–0.06; partial *r*=–0.03, not significant).

This indicates that the desire to avoid uncertainty and negative reactions to it are strongly associated with experiencing negative affective states because of health-related internet use, potentially because of increased catastrophic thinking about the meaning of bodily symptoms, as concluded by Fergus [21]. Another conceivable explanation for this finding may be that the tendency to react negatively to uncertainty and the resulting desire to avoid it may lead to further health-related internet use to reduce uncertainty. However, the more health-related internet uses are conducted, the greater the possibility of finding ambiguous and inconsistent information, which has the potential to trigger the negative affect.

In addition, intolerance of uncertainty was also found to moderate another detrimental effect of health-related internet use concerning anxiety sensitivity (see section *Anxiety Sensitivity*). Only individuals with high intolerance of uncertainty experience elevated anxiety sensitivity as a consequence of viewing web-based medical content [23].

##### Anxiety Sensitivity

Although people with intolerance of uncertainty experience uncertainty in general as dangerous, people with high levels of anxiety sensitivity interpret anxiety-related symptoms as dangerous [23]. This was found to be a potential risk factor for HA [87], and therefore, a link between anxiety sensitivity and cyberchondria-specific behavior was hypothesized [21,25]. It is commonly operationalized by the Anxiety Sensitivity Index-3 [88], which consists of three lower dimensions reflecting different types of concerns: *cognitive* (ie, concerns about mental incapacitations), *physical* (ie, concerns about immediate complications), and *social* (ie, concerns about social rejection because of publicly observable symptoms of anxiety). Correlational analyses supported the relevance of anxiety sensitivity for the cyberchondria construct (Multimedia Appendix 2). The coefficients range from a moderate to a high level. Different conclusions can be drawn from this study. The results may indicate that health-related internet uses are performed on various concerns (*cognitive*, *physical*, and *social*). Moreover, anxiety sensitivity may be relevant, especially for those experiencing distress because of health-related internet use. The structural equation models that included anxiety sensitivity, intolerance of uncertainty, and HA (operationalized by the SHAI) as independent variables to predict cyberchondria (operationalized by the CSS total score, excluding *mistrust*) highlighted the important role of anxiety sensitivity as a predictor for cyberchondria in addition to the contributions of intolerance of uncertainty and HA [25]. However, besides the role of anxiety sensitivity as a risk factor, it was also hypothesized to be a detrimental consequence of health-related internet use. Corresponding results are reported in detail in the section *Consequences of Health-Related Internet Use*.

##### Pain Catastrophizing

In the context of chronic pain, the contribution of pain catastrophizing to cyberchondria has been investigated [63]. It has been shown that pain catastrophizing (ie, the tendency to ruminate and worry about pain [89]) significantly predicts cyberchondria and its facets (operationalized by the CSS), even when controlling for negative affect and HA. Individuals with chronic pain often experience anxiety and distress about the origin and consequences of their pain perceptions and search for answers on the web. Pain catastrophizing may amplify the negative affect and may contribute to the initiation of the cyberchondria-specific vicious circle, as postulated by Starcevic and Berle [14]. Further support is provided by logistic regression analysis that aims to predict engaging in health-related internet use by psychopathologies and somatic symptoms [5] and comprises data from 992 adults from the general population of whom, 751 (75.7%) reported engaging in health-related internet uses in the past 3 months. It was shown that conducting health-related internet uses was associated with HA, obsessive-compulsive symptoms, and intolerance of uncertainty (among other psychopathologies); however, only the severity of somatic symptoms independently predicted health-related internet use when each of these pathological domains was controlled for.

#### Triggers for Health-Related Internet Use

Another relevant aspect in these models are the triggers for health-related internet use. For instance, Starcevic and Berle [14] postulated that health-related internet use is triggered by elevated levels of HA or distress. However, till now, research has paid little attention to the precursors, motivations, and triggers for health-related internet use. To our knowledge, only 2 qualitative studies have addressed this question, both using semistructured interviews with participants with high levels of HA (ie, participants’ SHAI scores exceeded a critical cutoff, indicating clinically significant HA (N=8, SHAI>16 points [90]; N=20, SHAI>18 points [1]). The studies yielded similar results, which are reported in the following section and integrated as far as possible with the superordinate themes identified by McManus et al [90] (*information is power* and *novelty of internet searching*). If no literature reference is given in parentheses, the 2 studies report the same results.

“*Information is power*” describes a participant’s hope to be able to prevent future illness, the deterioration of an already existing one [1], or death because of an illness [90] by gathering information, such as appropriate health promotion strategies or remedies [1]. In this context, Singh and Brown [1] found a positive association between HA and the frequency of using the internet to obtain *wellness* information. This can be interpreted as an attempt to prevent the onset of a serious disease, resulting in a feeling of being more in control of one’s health. The internet is also used for self-diagnoses to find a label for a health issue so that more specific information can be collected [90]. The possibility of failing to prevent an illness leads to the anticipation of dreadful consequences, which increases the perceived urgency to conduct health-related internet use [1]. Moreover, uncertainty about the probability of having a serious disease primed illness-related thoughts and worries leading to heightened anxiety, whereas being uncertain about nonserious issues led to curiosity [1]. In addition, health-related internet use is initiated by negative experiences and expectations concerning health care professionals that may lead to mistrust: inconvenience, too short appointment times, lack of reassurance, and past negative experiences. Participants also use the internet as a first filter for information to prepare for or justify a consultation with a physician.

The second superordinate theme was *novelty of internet searching* and included the perceived advantages of the internet as a medium of information compared with other methods. Advantages were a low threshold for use, the possibility to share others’ experiences and, in consequence, feel comforted by the knowledge that one is not the only person who suffers from this health issue [90], as well as speed, convenience, the possibility of obtaining opinions and advice from other persons affected, the huge size of the source, and its ability to provide reassurance. Reported disadvantages included the following: the information obtained was too broad, confusing, and often conflicting, as well as the presence of false, noncredible, or irrelevant information [1].

It can be concluded that a combination of different factors seems to lead to the initiation of health-related internet use. Besides situational factors (states) such as uncertainty and anxiety, more permanent or predisposing factors such as prior beliefs (eg, mistrust of medical professionals and *information is power*), seem to be important [1] as the metacognitive conceptualization of cyberchondria proposes [31]. There is still a lack of research investigating predisposing factors and triggers. Studies with naturalistic designs appear to be especially promising when investigating situational factors.

#### Consequences of Health-Related Internet Use

##### Emotional Consequences of Health-Related Internet Use

###### Negative Emotional Consequences of Health-Related Internet Use

The impact of health-related internet use on HA has mainly been assessed using questionnaires. Multimedia Appendix 9 [4,11,13,20,36,82] gives an overview of the results. The studies differ in terms of (1) the operationalizations of HA, health-related internet use, and its emotional effects; (2) the predictor for emotional effects (HA vs frequency of health-related internet use); and (3) the type of hypothesis and analysis (correlational vs difference). The results indicate that individuals with higher HA are more likely to experience negative emotional consequences after health-related internet use, which is in accordance with the study by Starcevic and Berle [14]. This finding is also supported by the results of the meta-regression that we conducted, as described in the section *A Meta-analytic Integration of Cyberchondria Operationalized by the CSS,* regarding the CSS subscale *distress* and its association with HA (β=0.66; *P*<.001). In addition, about one-third of individuals in general population samples stated that they felt increased anxiety after health-related internet use (31.4% [30]; 38.5% [91]). However, it should be noted that no measure of HA was included in this study.

To our knowledge, 2 experimental studies were conducted in this regard and supported the results of the questionnaire studies. Participants in a study by Baumgartner and Hartmann [13] read a text about a fictitious bacterial disease that is accompanied by ordinary symptoms such as stomach and intestinal pain. In addition, the trustworthiness of the source of information was manipulated. The information about the fictitious illness indeed increased negative responses in health-anxious individuals but only if the information was judged as trustworthy (perceived relevance of information: b=0.70, *P*<.01; chance of being already infected: b=0.94, *P*<.01; chance of getting infected: b=1.02, *P*<.01; and worry about disease: b=0.99, *P*<.01). Singh and Brown [37] showed that query escalations reduced postsearch anxiety immediately in 17.0% of the cases in the high HA group, remained the same in 27.65% of cases, and increased in 55.3% of cases. Proportions were comparable for the low HA group (χ²_2_=0.491; *P*=.78). Interview studies revealed that uncertainty, fright, anxiety, worry, and nervousness [1,90] could be experienced as a consequence of health-related internet use. Moreover, significant positive associations (*r*=0.31, *P*<.01 [19]; *r*=0.34, *P*<.001 [22]; *r*=0.44, *P*<.01 [63]) between cyberchondria (operationalized by the CSS total score) and negative affect (operationalized by the Positive and Negative Affect Schedule) were found, indicating that the higher the level of cyberchondria, more negative the affect experienced is. It should be noted that no statements can be made about a causal relationship.

Norr et al [23] hypothesized that being exposed to medical web-based information that is congruent with catastrophic interpretations of anxiety symptoms (namely, as a threat to health) may contribute to the development and maintenance of elevated anxiety sensitivity. They compared undergraduate students (N=52) in two conditions. The experimental group was exposed to medical websites converted to PDF files that provided catastrophic interpretations of bodily symptoms. The control group viewed the PDF files generated from general health and wellness websites. Hierarchical regression analyses showed that individuals in the experimental group experienced significantly higher anxiety sensitivity after the manipulation than did the control group (R^2^=0.81, *P*≤.001 and ∆R^2^=0.4, *P*≤.01). This relationship was moderated by the individuals’ levels of intolerance of uncertainty, such that viewing medical websites only affected anxiety sensitivity in participants with high levels of intolerance of uncertainty. Therefore, it was concluded that health-related internet use and the concomitant exposure to medical content might call users’ attention to potentially alarming causes of bodily sensations and may thereby fuel both HA and anxiety sensitivity [21,25].

###### Positive Emotional Consequences of Health-Related Internet Use

Besides the reported results confirming the postulated negative emotional consequences of health-related internet use, it should be noted that positive emotional consequences were also found, such as relief and calm [1,11,90]. The process of searching itself can provide reassurance by giving a person the feeling of having done something instead of ruminating [90]. As mentioned above, Singh and Brown [37] found that in escalated health-related internet use, anxiety after search was reduced in 17.0% of cases, even in individuals with high levels of HA. Doherty-Torstrick et al [82] found that a considerable proportion of their sample (32.8% in the high HA group and 71.2% in the low HA group) experienced no impact or decreased anxiety during and after health-related internet use. Fergus and Dolan [30] stated that 40.7% of their sample experienced reduced HA after health-related internet use. White and Horvitz [91] reported that 76% of their participants felt reassured, and 50.3% experienced reduced anxiety.

###### Predictors of Emotional Consequences of Health-Related Internet Use

Some of the factors influencing whether positive or negative emotional consequences are more likely experienced have already been discussed above (ie, HA, intolerance of uncertainty, anxiety sensitivity, and obsessive-compulsive symptoms). However, certain characteristics of the searching process itself also seem to have an influence. Substantial associations between the frequency and the duration of searches and elevated HA postsearch were found (Multimedia Appendix 9). Individuals engaging more often or longer in this behavior are more likely to experience negative consequences. McManus et al [90] found that the longer individuals searched the web, the more likely they were to find alarming information that, in turn, would lead to heightened anxiety. Another important factor seems to be the type of information found: participants stated that negative emotions occurred during health-related internet use if unfamiliar health issues were researched or potentially alarming information was found [1,90]. Conversely, positive emotions were experienced if familiar issues were researched or nonserious causes for experienced symptoms were found. Postsearch negative emotions occurred when people perceived the information as inconclusive, conflicting, confusing, or serious. In this case, new searches were initiated. Postsearch positive emotions were the result of finding a nonserious answer, diagnosing oneself, finding a remedy, or clarifying uncertainties. In these cases, the search was terminated. The results regarding query escalations support these findings [6,37]. Furthermore, the type of web-based service used seems to have an impact: using forums that provide web-based contact with experts or the possibility of sharing with other affected persons seems to alleviate anxiety, whereas using diagnosis systems and video platforms seems to have the opposite effect [36].

###### Reciprocity Between Health-Related Internet Use and HA

TePoel et al [81] investigated whether HA influences health-related internet use and vice versa. They chose a -wave longitudinal study design with 2-month time lags between the waves. Health-related internet use was operationalized by frequency. Analyses were conducted separately for individuals with lower levels of HA (N=4,564, Dutch version SHAI score<18) and pathological levels of HA (N=751, Dutch version SHAI score≥18); this is of particular importance regarding the distinctiveness of cyberchondria from HA and the question of whether the mechanisms underlying cyberchondria are different in individuals with different levels of HA. A random intercept cross-lagged panel model was chosen for the data analysis. This multilevel approach is able to control for stable (trait-like) individual differences by distinguishing between within-level and between-level variances. In this model, two random intercepts were included for HA and health-related internet use. In the pathological HA group, changes in an individual’s HA score were not predicted by a change in health-related internet use 2 months earlier. Conversely, changes in an individual’s health-related internet use score were not predicted by changes in HA 2 months earlier. Thus, no evidence for a reciprocal relationship between HA and health-related internet use was found in individuals with pathological HA. In contrast, evidence for a reciprocal association was found in the nonclinical group with lower HA, such that increases in an individual’s HA were predicted by increases in health-related internet use and vice versa.

This indicates that the underlying mechanisms for the impact of health-related internet use on HA differ depending on the previously existing level of HA. In the pathological HA group, health-related internet use seemed to be a maintaining rather than an increasing factor. This suggests that findings from samples with normal levels of HA should only be transferred to clinical samples with care. This highlights the importance of investigating the whole range of HA (especially clinical samples) and its immediate effects. The question of causality remains unanswered in this context.

##### Effects of Health-Related Internet Use on Behavior: Reassurance Seeking and Health Care Use

It has been hypothesized that health-related internet use is able to trigger further health care use to receive reassurance, especially in individuals with elevated HA. This hypothesis is supported by several findings. Singh and Brown [11] found a medium correlation between HA and the likelihood of visiting a physician after the search (*r*=0.22; *P*<.01). Our meta-regression (see section *A Meta-analytic Integration of Cyberchondria Operationalized by the CSS*) regarding the association between the CSS subscale *reassurance* and HA yielded a strong correlation (β=.44; *P*<.001). Eastin and Guinsler [92] investigated the moderating role of HA in this process. HA was operationalized by the Health Anxiety Questionnaire [93], health-related internet use by its frequency, and health care use because of health-related internet use by the frequency of physician consultations and the number of different physician visits. Regression analysis confirmed a significant interaction, indicating that as HA increases, the relationship between the frequency of health-related internet use and health care use increases. Furthermore, individuals with high HA (Illness Attitude Scale≥47 [41]) considered dysfunctional behavior (eg, physician hopping and ordering nonprescribed medication on the web) to be more likely because of health-related internet use [36]. In terms of the Cyberchondria model proposed by Brown et al [26], these results might indicate that individuals with normal levels of HA are better able to correctly judge the seriousness of the threat originating from health-related internet use and decide whether a physician consultation is necessary. Interestingly, it seems as if the use of physical health care plays a more important role compared with mental health care use. Using structural equation modeling, the CSS subscale *reassurance* showed a stronger association with the use of physical health care (β=.70) than with mental health care (β=.50) over the past 60 days [64]. In addition, the subscale *excessiveness* was negatively associated with mental health care use (β=–.49*; P*<.001), indicating that individuals who repeatedly engage in health-related internet use report less use of mental health care.

##### Effects of Health-Related Internet Use: Quality of Life and Functional Impairment

In an extension of the *compulsion* subfacet of cyberchondria [17], which reflects different ways in which health-related internet use can interrupt other web-based and offline activities, it was hypothesized that cyberchondria might lead to significant impairment in psychosocial functioning.

In line with this, it was found that cyberchondria was strongly associated with greater functional impairment even when controlling for the effects of HA (operationalized by the SHAI [68]) [64]. Functional impairment was defined as one’s ability to engage in daily activities, operationalized using the Sheehan Disability Scale [94]. Interestingly, when accounting for HA, CSS was not associated with decreased quality of life, which was operationalized as one’s overall level of contentment and satisfaction regarding 4 domains: physical and psychological health, social relationships, and environment (World Health Organization Quality of Life Assessment [95]). These results might indicate that individuals with cyberchondria may be satisfied with their lives, although they feel functionally impaired by their cyberchondria-specific behavior.

## Discussion

### Principal Findings

**Figure 5:**
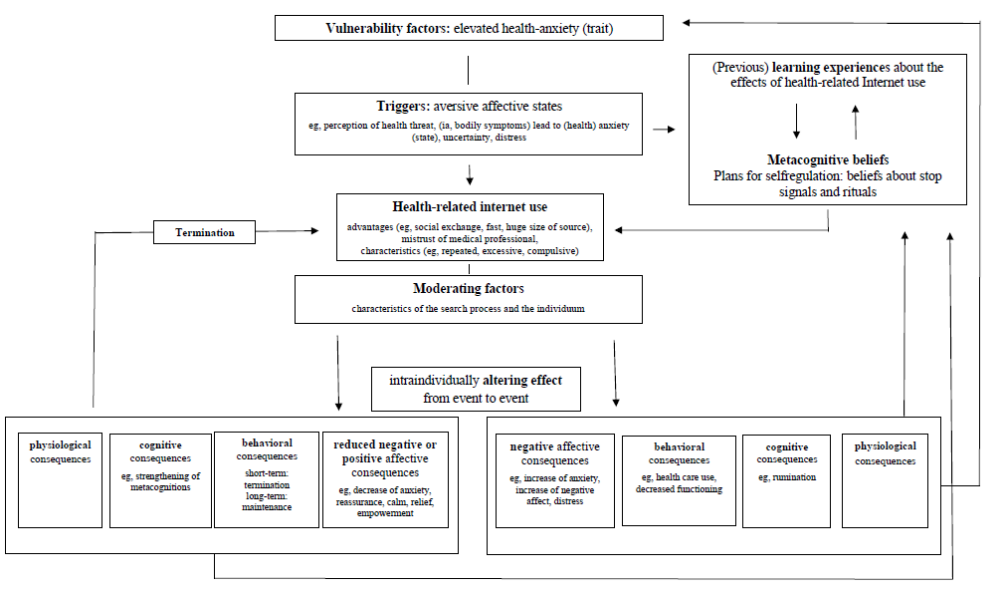
Hypothetical cognitive-behavioral model of cyberchondria as a health-related safety behavior maintained through intermittent reinforcement.

The aims of this work were to (1) give an overview of existing conceptualizations of cyberchondria and its relation to anxiety-related pathologies, (2) quantify its association with HA, and (3) highlight gaps in the current literature. In an attempt to summarize and reconcile the partly contradictory models and findings, an integrative hypothetical cognitive-behavioral model of cyberchondria as a health-related safety behavior that is maintained through intermittent reinforcement was developed (aim 4; Figure 5).

Previous conceptualizations of cyberchondria proposed that health-related internet use should be divided into *cyberchondria* and *classic safety behavior* based on its (emotional) impact. However, in the context of pathological HA, there is no evidence that health-related internet use followed by negative emotional consequences is conceptually different from health-related internet use followed by positive ones. This distinction marks the crucial point regarding the incompatibility of definitions and the integration of existing findings on emotional consequences. Regarding elevated levels of HA, we, therefore, propose the conceptualization of cyberchondria as a classic health-related safety behavior that is maintained by intermittent reinforcement. In addition, following Brown et al [26], we recommend not to use the term *cyberchondri*a any longer, inter alia, as this would imply a conceptual distinction between health-related internet use and other health-related safety behaviors. In the following, *cyberchondria* is used only to mark results that were assessed using previous conceptualizations (eg, the CSS). In the following, the components of our model are explicated in detail.

All existing theories suggest that cyberchondria-specific behavior is triggered by emotions that can be summarized as aversive emotional states. A more recent model [26] suggests that the perception of a health threat may be central to initiation. We also share this view in the field of elevated and pathological HA. Confronted with *situations* that are evaluated as threatening to health, highly health-anxious individuals are hypothesized to experience, for example, (health) anxiety, uncertainty, and distress. Such a situation may be, for instance, viewing illness-related materials or even the perception of bodily symptoms. As is known for pathological HA, there is also support for the view that this factor may be relevant. To mention, there are the substantial associations between cyberchondria and health concerns triggered by bodily sensations (as assessed with the Whiteley Index), anxiety sensitivity, and the severity of somatic problems (as seen in the context of chronic pain). However, to our knowledge, no study exists that examines the perception of bodily symptoms as a trigger.

Besides the emotional aspects, the perception of a health threat may have consequences on the cognitive (eg, health concerns) and physiological levels (ie, changes corresponding to the affective state). Regarding the latter point, the process of somatosensory amplification [59] may contribute to the initiation and maintenance of health-related internet use. At the behavioral level, health-related internet use may be conducted to reduce (concordant) negative affect. Interview studies give the first indications that health-related internet use is triggered by a combination of stable and situational factors, supporting the conceptualization of cyberchondria in a cognitive-behavioral model. To our knowledge, no studies have addressed the important question of immediate precursors (especially state HA) in an experimental or naturalistic study design.

The first existing questionnaire-based studies highlighted the relevance of metacognitions regarding cyberchondria-specific behavior. Moreover, the reported results of interview studies contained results that can be integrated into this context (eg, *Information is power*). In terms of content, metacognitions are usually distinguished as positive and negative with distinct functions (initiation vs maintenance of health-related internet use); however, the results are heterogeneous. Therefore, this separation was not included in our model. Following Fergus and Spada [22,31] as well as Brown et al [26], we also propose for the field of elevated and pathological HA that aversive negative affective states activate metacognitive beliefs that may represent plans for self-regulation strategies (namely health-related internet use), rituals, and stop signals that are conducted to cope with aversive (affective) states. At this point, we expand existing theories by supplementing the interaction between metacognitions and previous learning experiences regarding the (short-term emotional) consequences of health-related internet use. The latter may include the experience that health-related internet use may have altering effects from event to event; that is, that besides negative emotions, positive consequences (eg, relief) can also occur. Therefore, on the one hand, metacognitions may develop from learning experiences (eg, “HIU is useful. At some point, I will feel better”). On the other hand, these metacognitions may influence how negative outcomes of health-related internet uses are processed and contribute to its maintenance.

Health-related internet use seems to have certain advantages compared with other health-related safety behaviors that may make it more likely for health-anxious individuals to select this strategy over others. Besides obvious aspects (ie, anonymity, huge amount of specific information, low costs, and promptness), social contact and exchange seem to be relevant. Regarding mistrust of professionals, partly contradictory results were reported, and the general recommendation of not to include this aspect in the construct of cyberchondria was critically discussed. Brown et al [26] included this factor as previous experiences in their model as a kind of vulnerability factor. We also see sufficient support for this but interpret mistrust in the context of why health-related internet use is selected over other safety behaviors. In addition, it is conceivable that this aspect may be more relevant in samples with pathological levels of HA, given that the relationships between physicians and highly health-anxious patients are often difficult and characterized by dissatisfaction [96].

In addition, numerous results exist regarding the characteristics of health-related internet uses once the vicious circle process is set into motion. The reported findings support the notion that health-related internet use occurs repeatedly and has excessive and compulsive subcomponents. Individuals with higher HA engage in health-related internet use for longer periods and more frequently (Multimedia Appendix 7). Moreover, they are more likely to repeat searches and use numerous sources, as well as to experience that health-related internet use interrupts other activities. Correlational and factor analysis examinations yielded further evidence (especially regarding the positive association between HA and the CSS subfacets, *excessiveness* and *compulsion*). Concerning compulsive elements, further supportive results were found: first, an association between HA and the 6 indicators of addiction to health-related internet use, and second, a strong association between problematic internet use (reflecting behaviors from the fields of compulsion and addiction) and cyberchondria.

Concerning the consequences of health-related internet use, all authors agree that immediately after health-related internet use, both positive emotional consequences (especially reduction of HA), as well as negative ones (increase of HA) can occur. Indeed, the existence of both valences was confirmed even in highly health-anxious individuals. Most previous studies have focused on interindividual differences concerning emotional consequences in cross-sectional study designs. With this approach, the effects of health-related internet use are indirectly assumed to be stable over time within an individual (almost trait-like); however, this has not yet been investigated. Future studies should follow an event-based approach to investigate possible changes in intraindividual effects over time. If individuals experience different effects of health-related internet use, altering from event to event, this may be an indicator of the presented conceptualization.

Previous findings suggest that individuals with higher degrees of anxiety-related pathologies (obsessive-compulsive symptoms, intolerance of uncertainty, and anxiety sensitivity) are more likely to experience cyberchondria, especially negative emotional consequences (Multimedia Appendix 9). Following TePoel et al [81], we hypothesize that an illness-related bias may be of certain relevance regarding the moderating role of emotional outcomes. It was found to occur in highly health-anxious individuals [97,98], as well as in patients with pathological HA [99-101] and describes the tendency to focus on information that confirms their health worries and to ignore information that is contradictory [58]. However, to our knowledge, no study has addressed this bias in the context of cyberchondria. In addition, the characteristics of the medical search session itself seem to influence emotional outcomes. The type of information found may also be relevant (alarming and unfamiliar and *query escalation*), as well as the greater frequency and longer duration that is more likely to produce elevated negative emotional states (Multimedia Appendix 9). The results regarding the moderating role of the perceived trustworthiness of a source are inhomogeneous and therefore not included in the model. We also propose the consequences of health-related internet use on other behavioral components corresponding to the affective outcome, as shown in Figure 5. As the results indicate that bodily symptoms may be a trigger, it seems conceivable that they increase and decrease corresponding to the emotional effects of health-related internet use. Cognitive and physiological consequences need to be further investigated.

We hypothesize that health-related internet use is maintained through intermittent reinforcement (ie, altering valences of effects). For the positive valence effects, we propose two possible ways of maintenance. First, through negative reinforcement, that is, in consequence of health-related internet use, aversive states may be reduced, such as the perceived health threat, concordant negative feelings (eg, HA), or aversive physiological (anxiety) symptoms. Second, through positive reinforcement, positive valence emotions may occur (eg, reassurance, relief, calm, empowerment, and increased sense of control). Regarding negative valence consequences, the above-described metacognitive beliefs (“Some time or other, I will feel better”) may maintain health-related internet use besides its negative effects. In addition, it seems conceivable that amplification of aversive states may retrigger self-regulative behavior (ie, health-related internet use).

### Strengths and Limitations

The strengths of this review include the updated systematic research strategy based on the PRISMA guidelines and the focus on the models underlying the studies. All potentially eligible studies were assessed for inclusion. The meta-analyses make an important contribution to this field by aggregating the results of numerous studies and quantifying the association between cyberchondria and HA. The development of a hypothetical CBT model for elevated and pathological HA provides further starting points for future research and treatment. There were also limitations of the review. Although trying to make the literature search as inclusive as possible, the exclusion criteria may have resulted in the omission of relevant studies. Potential papers not written in English or German or those investigating children and adolescents were excluded, which may have resulted in linguistic, cultural, or age bias. Moreover, the generalizability of the meta-analytic results to the level of constructs is restricted: first, because of the inclusion of relatively few studies and the operationalization of cyberchondria and HA, and second, because samples were mainly recruited via the internet. This may lead to an artificially heightened homogeneity of samples, which restricts the search for moderators.

### Conclusions

Cyberchondria-specific behaviors appear distinct from but strongly related to HA, intolerance of uncertainty, obsessive-compulsive symptoms, and anxiety sensitivity. Numerous findings support the dominant conceptualization of cyberchondria by Starcevic and Berle [14], which postulates a bidirectional relationship between health-related internet use and HA. However, no clear evidence exists for the key elements of previous definitions regarding the conceptual differentiation of cyberchondria as a classic safety behavior based on its emotional consequences. Future research needs to further investigate the immediate emotional consequences of health-related internet use, especially in individuals with pathological HA on an interindividual level, using experimental and naturalistic longitudinal study designs. Additional variables besides HA (eg, feelings of uncertainty) have to be taken into account when examining the mechanisms of initiation and maintenance that are mediated by the immediate effects of health-related internet use.

## Appendix

Multimedia Appendix 1 Correlations between intolerance of uncertainty and cyberchondria.

Multimedia Appendix 2 Correlations between anxiety sensitivity and cyberchondria.

Multimedia Appendix 3 Summary of currently existing conceptualizations of cyberchondria and their key elements.

Multimedia Appendix 4 Factor intercorrelations and internal consistencies of the Cyberchondria Severity Scale.

Multimedia Appendix 5 Correlations between health anxiety and the Cyberchondria Severity Scale subscales.

Multimedia Appendix 6 Quality ratings of integrated studies.

Multimedia Appendix 7 Association between health anxiety and cyberchondria operationalized by frequency and duration of health-related internet use.

Multimedia Appendix 8 Correlations between obsessive-compulsive symptoms and the Cyberchondria Severity Scale subscales.

Multimedia Appendix 9 Emotional effects of health-related internet use.

## Abbreviations

CBT: cognitive-behavioral therapy
CSS: Cyberchondria Severity Scale
DOCS: Dimensional Obsessive-Compulsive Scale
HA: health anxiety
MCQ-HA: Metacognitions Questionnaire–Health Anxiety
PRISMA: Preferred Reporting Items for Systematic Reviews and Meta-Analyses
SHAI: Short Health Anxiety Inventory
