# Supplementary material for: Conceptualizations of Cyberchondria and Relations to the Anxiety Spectrum: Systematic Review and Meta-analysis
Source: J Med Internet Res. 2021 Nov 18;23(11):e27835. doi: 10.2196/27835 (PMC8663695; doi:10.2196/27835)
Supplement: Multimedia Appendix 1 [file jmir_v23i11e27835_app1.docx]

**Table A.1. Correlations between intolerance of uncertainty and cyberchondria.** * = *P* < .05. ** = *P* < .001. HIU = health-related Internet use. CSS = Cyberchondria Severity Scale. IUS-12 = Intolerance of Uncertainty Scale.

| **First author, year** | **Operationalization of intolerance of uncertainty** | **Operationalization of**  **cyberchondria** | **Pearson correlation coefficient *r*** |
| --- | --- | --- | --- |
| Fergus, 2013 [20] | IUS-12  Total score | Frequency of HIU | .33* |
| Fergus, 2013 [20] | IUS-12  Total score | HA in response to HIU | .30* |
| Norr et al., 2014 [23] | IUS-12  Prospective scale | CSS total scale (excluding *Mistrust*) | .38* |
| Norr et al., 2014 [23] | IUS-12  Inhibitory scale | CSS total scale (excluding *Mistrust*) | .50* |
| Fergus, 2015 [21] | IUS-12  Inhibitory scale | CSS total scale  *Compulsion*  *Distress*  *Excessiveness*  *Reassurance* | .47**  .39**  .49**  .40**  .24** |
| Fergus, 2015 [21] | IUS-12  Prospective scale | CSS total scale  *Compulsion*  *Distress*  *Excessiveness*  *Reassurance* | .33**  .17**  .33**  .36**  .18** |
| Fergus & Spada, 2018, Study 1 [22] | IUS-12  Inhibitory scale | CSS total scale (excluding *Mistrust*) | .31** |
|  | IUS-12  Prospective scale | CSS total scale (excluding *Mistrust*) | .24** |
