# Supplementary material for: Conceptualizations of Cyberchondria and Relations to the Anxiety Spectrum: Systematic Review and Meta-analysis
Source: J Med Internet Res. 2021 Nov 18;23(11):e27835. doi: 10.2196/27835 (PMC8663695; doi:10.2196/27835)
Supplement: Multimedia Appendix 2 [file jmir_v23i11e27835_app2.docx]

**Table A.2. Correlations between anxiety sensitivity and cyberchondria.** * = *P* < .05. ** = *P* < .001. CSS = Cyberchondria Severity Scale. ASI-3 = Anxiety Sensitivity Index-3.

| **First author, year** | **Operationalization of anxiety sensitivity** | **Operationalization of**  **cyberchondria** | **Pearson correlation coefficient *r*** |
| --- | --- | --- | --- |
| Fergus, 2015 [21] | ASI-3: *Cognitive* | CSS total scale  *Compulsion*  *Distress*  *Excessiveness*  *Reassurance* | .55**  .53**  .53**  .37**  .35** |
|  | ASI-3: *Physical* | CSS total scale  *Compulsion*  *Distress*  *Excessiveness*  *Reassurance* | .55**  .46**  .59**  .40**  .33** |
|  | ASI-3: *Social* | CSS total scale  *Compulsion*  *Distress*  *Excessiveness*  *Reassurance* | .40**  .29**  .39**  .38**  .21** |
| Norr, Albanese et al., 2015 [25] | ASI-3 total score | CSS total scale  (excluding *Mistrust*) | .57* |
| Fergus & Spada, 2018, Study 1 [22] | ASI-3: *Cognitive* | CSS total scale  (excluding *Mistrust*) | .44** |
|  | ASI-3: *Physical* | CSS total scale  (excluding *Mistrust*) | .37** |
|  | ASI-3: *Social* | CSS total scale  (excluding *Mistrust*) | .35** |
