# Supplementary material for: Conceptualizations of Cyberchondria and Relations to the Anxiety Spectrum: Systematic Review and Meta-analysis
Source: J Med Internet Res. 2021 Nov 18;23(11):e27835. doi: 10.2196/27835 (PMC8663695; doi:10.2196/27835)
Supplement: Multimedia Appendix 3 [file jmir_v23i11e27835_app3.docx]

**Table A.3. Summary of currently existing conceptualizations of cyberchondria and their key elements.** HA = health anxiety. HIU = health-related Internet use. ↑ = short-term increase of negative affect. ↓ = short-term decrease of negative affect.

| **Authors, year** | **Conceptualization** | **Precursors** | **Immediate effects of HIU** | | **Mechanism of maintenance** | **Background** |
| --- | --- | --- | --- | --- | --- | --- |
| White & Horvitz, 2009 [6] | search characteristics: query escalation | concerns, anxiety | unfounded escalation of concerns about common symptomatology | ↑ | search process characteristics, judgment biases (base rate neglect, availability bias) | review of empirical findings |
| Starcevic & Berle, 2013 [14] | failed safety behavior | distress, anxiety | increase in distress and anxiety | ↑ | search process characteristics, information overload, need for perfect explanation, uncertainty, trustworthiness of sources | review of empirical findings |
| Fergus & Dolan, 2014 [30] | a form of problematic Internet use | negative emotions | increase in distress | ↑ | abundance of health information on the Internet, rumination | cognitive–behavioral models of pathological Internet use by Davis (2001) |
| Bleichhardt & Weck, 2015 [15] | safety behavior | increased negative affect (e.g., anxiety, HA, worry) | decrease in negative affect | ↓ | negative reinforcement | cognitive-behavioral model of health anxiety and hypochondriasis by Warwick & Salkovskis (1990) |
| Fergus & Spada, 2017 [31] and 2018 [22] | metacognitive beliefs about HIU as a self-regulation process | aversive emotional states | increase in distress | ↑ | initiation and maintenance of HIU because of metacognitive beliefs | metacognitive model of emotional disorders by Wells & Matthews (1996) |
| Brown et al. (2020) [26] | safety behavior | health threat | two possible outcomes: reassurance or anxiety | ↓/↑ | maintenance of HIU because of metacognitive beliefs | review of empirical findings; hybrid model: combines reassurance, compulsive and meta-cognitive elements |
