# Supplementary material for: Conceptualizations of Cyberchondria and Relations to the Anxiety Spectrum: Systematic Review and Meta-analysis
Source: J Med Internet Res. 2021 Nov 18;23(11):e27835. doi: 10.2196/27835 (PMC8663695; doi:10.2196/27835)
Supplement: Multimedia Appendix 4 [file jmir_v23i11e27835_app4.docx]

**Table A.4. Factor intercorrelations and internal consistencies of the Cyberchondria Severity Scale.** a = McElroy & Shevlin (2014) [17]. b = Fergus (2014) [16]. c = Norr et al. (2015) [18]. d = Barke et al. (2016) [39]. e = Fergus & Russell (2016) [19]. f = Mathes et al. (2018) [41]. g = Bajcar et al. (2019) [40]. Total Scale R = Total Scale Revised, i.e., without Mistrust factor. ** = *P* < .01 (b: two-tailed, c and d: not reported). * = *P* < .05 (b: two-tailed, c and d: not reported). Cronbach's alpha coefficients listed in parentheses. No significances were reported in a.

|  | Compulsion | |  |  |  |  |  | Distress | |  |  |  |  |  | Excessiveness | | |  |  |  |  |
| --- | --- | --- | --- | --- | --- | --- | --- | --- | --- | --- | --- | --- | --- | --- | --- | --- | --- | --- | --- | --- | --- |
| Study | a | b | c | d | e | f | g | a | b | c | d | e | f | g | a | b | c | d | e | f | g |
| Compulsion | (.95) | (.95) | - | (.90) | - | (.96) | (0.88) |  |  |  |  |  |  |  |  |  |  |  |  |  |  |
| Distress | .56 | .68** | .80* | .59** | .61** | .75* | 0.56** | (.92) | (.95) | - | (.87) | - | (.95) | (.92) |  |  |  |  |  |  |  |
| Excessiveness | .54 | .46** | .63* | .44** | .42** | .54* | 0.54** | .67 | .62** | .72* | .55** | .60** | .61* | 0.67** | (.85) | (.87) | - | (.74) | - | (.87) | (.87) |
| Reassurance | .54 | .52** | .65* | .26** | .44** | .50* | 0.54** | .56 | .54** | .66* | .43** | .45** | .56* | 0.56** | .52 | .60** | 67* | .45** | .55** | .59* | 0.52** |
| Mistrust | .23 | .23** | <.01 | .21** | - | - | 0.23** | .17 | .14** | .03 | .12 | - | - | 0.17* | .13 | .01 | .16* | .21** | - | - | 0.13* |
| Total Scale | - | .82** | - | * | - | - | - | - | .88** | - | .85** | - | - | - | - | .80** | - | .80** | - | - | - |
| Total Scale R | - | .81** | - | - |  | - | - | - | .88** | - | - | .86** | - | - | - | .82** | - | - | .82** | - | - |
|  |  |  |  |  |  |  |  |  |  |  |  |  |  |  |  |  |  |  |  |  |  |
|  |  |  |  |  |  |  |  |  |  |  |  |  |  |  |  |  |  |  |  |  |  |
|  | Reassurance | |  |  |  |  |  | Mistrust | |  |  |  |  |  |  |  |  |  |  |  |  |
| Study | a | b | c | d | e | f | g | a | b | c | d | e | f | g |  |  |  |  |  |  |  |
| Compulsion |  |  |  |  |  |  |  |  |  |  |  |  |  |  |  |  |  |  |  |  |  |
| Distress |  |  |  |  |  |  |  |  |  |  |  |  |  |  |  |  |  |  |  |  |  |
| Excessiveness |  |  |  |  |  |  |  |  |  |  |  |  |  |  |  |  |  |  |  |  |  |
| Reassurance | (.89) | (.88) | - | (.76) | (.88) | (.85) | (.80) |  |  |  |  |  |  |  |  |  |  |  |  |  |  |
| Mistrust | -.04 | - .04 | 28* | .03 | - | - | -.04 | (.75) | (.87) | - | (.89) | (.84) | - | (.62) |  |  |  |  |  |  |  |
| Total Scale | - | .75** | - | .65** | - | - | - | - | .26** | - | .34** | - | - | - |  |  |  |  |  |  |  |
| Total Scale R | - | .78** | - | - | .73** | - | - | - | .11* | - | - | - | - | - |  |  |  |  |  |  |  |
