# Supplementary material for: Conceptualizations of Cyberchondria and Relations to the Anxiety Spectrum: Systematic Review and Meta-analysis
Source: J Med Internet Res. 2021 Nov 18;23(11):e27835. doi: 10.2196/27835 (PMC8663695; doi:10.2196/27835)
Supplement: Multimedia Appendix 5 [file jmir_v23i11e27835_app5.docx]

**Table A.5. Correlations between HA and the Cyberchondria Severity Scale subscales.** HA = health anxiety. CSS = Cyberchondria Severity Scale. SHAI = Short Health Anxiety Inventory. MIHT = Multidimensional Inventory of Hypochondriacal Traits. * = *P* < .05, ** = P < .01. ^1^ The CSS-15 was used. ^2^ The same sample was analyzed as by Norr, Albanese et al. (2015) [23].

| **Study** | **HA** | | | **CSS** | | | | | |
| --- | --- | --- | --- | --- | --- | --- | --- | --- | --- |
|  |  |  |  | **Compulsion** | **Distress** | **Excessiveness** | **Reassurance** | **Mistrust** | **CSS Total (without Mistrust)** |
| Norr, Oglesby et al. (2015) [18] | SHAI | | | .49** | .60** | .51** | .39** | - | - |
| Barke et al. (2016) [39] | mSHAI | | | .42** | .85** | .80** | .65** | .34** | - |
| Barke et al. (2016)^1^ [39] | mSHAI | | | .39** | .73** | .76** | .66** | .30** | - |
| Norr, Allan, Boffa, Raines, & Schmidt (2015)^2^ [38] | SHAI | Thought Intrusion | | .38* | .49* | .46* | .34* | .01 | - |
|  |  | Fear of Illness | | .43* | .52* | .43* | .36* | -.08 | - |
| Fergus & Russell (2016) [19] | MIHT | affective | | .41** | .62** | .46** | .27** | - | .57** |
|  |  | perceptual | | -.05 | .08 | .28** | .09 | - | .13* |
|  |  | cognitive | | .41** | .46** | .33** | .22** | - | .46** |
|  |  | behavioral | | .15** | .30** | .32** | .22** | - | .32** |
| Mathes et al. (2018) [64] | SHAI | Thought  Intrusion | | .37* | .49* | .45* | .34* | - | - |
|  |  | Fear of Illness | | .41* | .52* | .44* | .33* | - | - |
| Gibler et al. (2019) [63] | SHAI | | | .50** | .55** | .53** | .43** | - | .58** |
| Bajcar et al. (2019) [40] | SHAI | | HA | .50** | .59** | .45** | .36** | .08 | .56** |
|  |  |  | Illness Likelihood | .49** | .56** | .45** | .36** | .07 | .52** |
|  |  |  | Negative Consequences of Illness | .31** | .39** | .23** | .19** | .06 | .33** |
