# Supplementary material for: Conceptualizations of Cyberchondria and Relations to the Anxiety Spectrum: Systematic Review and Meta-analysis
Source: J Med Internet Res. 2021 Nov 18;23(11):e27835. doi: 10.2196/27835 (PMC8663695; doi:10.2196/27835)
Supplement: Multimedia Appendix 6 [file jmir_v23i11e27835_app6.docx]

**Table A.6. Quality ratings of integrated studies.**  nsc = not stated clearly.

|  | **Sample size**  ***N* ≥ 85** | **Consecutive sampling** | **Sufficiently reported inclusion/exclusion criteria** | **Quality items** | **Control for potentially confounding variables** | **Sufficiently reported sample characteristics** | **Representativeness** | **Overall rating** |
| --- | --- | --- | --- | --- | --- | --- | --- | --- |
| **First author, year** |  |  |  |  |  |  |  |  |
| Fergus, 2014 [16] | yes | yes | yes | yes | yes | yes | yes | high |
| Fergus, 2015 [21] | yes | yes | yes | no | yes | yes | yes | high |
| Norr, Albanese et al., 2015 [25] | yes | yes | yes | yes | yes | yes | yes | high |
| Barke et al., 2016, Study A [39] | yes | nsc | yes | yes | no | no | yes | medium |
| Barke et al., 2016, Study B [39] | yes | nsc | yes | yes | no | no | yes | medium |
| Fergus & Russell, 2016 [19] | yes | yes | yes | no | yes | yes | yes | high |
| Fergus & Spada, 2017, Study 2 [31] | yes | yes | yes | no | yes | yes | yes | high |
| Fergus & Spada, 2018, Study 1 [22] | yes | yes | no | yes | yes | yes | no | medium |
| Fergus & Spada, 2018, Study 2 [22] | yes | yes | yes | yes | yes | yes | yes | high |
| Bajcar et al., 2019 [40] | yes | nsc | no | no | no | yes | yes | low |
| Gibler et al., 2019 [63] | yes | yes | yes | yes | yes | yes | no | high |
| Mathes et al., 2019 [64] | yes | yes | yes | yes | yes | yes | yes | high |
