# Supplementary material for: Conceptualizations of Cyberchondria and Relations to the Anxiety Spectrum: Systematic Review and Meta-analysis
Source: J Med Internet Res. 2021 Nov 18;23(11):e27835. doi: 10.2196/27835 (PMC8663695; doi:10.2196/27835)
Supplement: Multimedia Appendix 7 [file jmir_v23i11e27835_app7.docx]

**Table A.7. Association between HA and cyberchondria operationalized by frequency and duration of HIU.** HA = health anxiety. HIU = health-related Internet use. In: Performing HIU = inclusion criterion: performing HIU. Ex: Medical Condition = exclusion criterion: suffering from a diagnosed medical condition. WI = Whiteley Index. SHAI = Short Health Anxiety Inventory. IAS = Illness Attitude Scale. DSHAI = Dutch version of the SHAI. When interpreting the results by Muse et al. (2012) [4] it should be noted, that a Pearson or Spearman correlation coefficient *r* equals the regression coefficient β in an unadjusted analysis.

| **First authors, year** | **Sample** | **Operationalization** | | **Results: Association between HA and HIU** | **Country** | **Age**  *M* (*SD*) | **In: Per-forming HIU** | **Ex: Medical Condition** |
| --- | --- | --- | --- | --- | --- | --- | --- | --- |
|  |  | **HA** | **HIU** |  |  |  |  |  |
| Baumgartner & Hartmann, 2011 [13] | *N* = 104  under- / postgraduates | WI | frequency | β = .46, *P* < .01 | Nether-lands | 21.02  (2.19) | no | no |
| Muse et al., 2012 [4] | *N* = 167  *n* = 46 high HA  *n* = 36 low HA  mostly students | SHAI | frequency  (5-point Likert scale) | β = 0.06, *P* < .001 (stepwise linear regression) | Great Britain | not reported | yes | no |
|  |  |  |  | low HA *M*[*SD*] = 0.17 [0.38] vs.  high HA *M* [*SD*] = 1.30 [1.19], *t*(80) = 5.52, *P* < .001 |  |  |  |  |
|  |  |  | duration  (in minutes) | β = 0.43, *P* < .01 (stepwise linear regression) |  |  |  |  |
|  |  |  |  | low HA *M*[*SD*] = 17.43 [11.41] vs.  high HA *M* [*SD*] = 26.78 [20.41], *F*(1,79) = 3.88, *P* < .05 |  |  |  |  |
| Fergus, 2013 [20] | *N* = 454  general population | SHAI | frequency | *r* = .48, *P* < .01 | USA | 33.6  (11.9) | yes | yes |
| Singh & Brown, 2014 [11] | *N* = 255  under-/ postgraduates | SHAI | frequency | *r* = .16, *P* < .01 | Great Britain | 21.24  (3.92) | yes | no |
|  |  |  | duration | *r* = .17, *P* < .01 |  |  |  |  |
| Doherty- Torstrick et al., 2016 [82] | *N* = 720  general population  *n* = 640 high HA (WI > 30);  *n* = 80 low HA (WI ≤ 30) | WI | duration  (most time/ day in the past month) | low HA *M*[*SD*] = 1.30 [0.49] “1-3 hours” vs. high HA *M* [*SD*] = 2.28 [0.93] “less than 1 hour”, *t*(718) = -9.13, *P* < .01, *d* = 1.10 | USA | 33.01  (12.08) | yes | no |
|  |  |  | frequency  (worst day/past year) | low HA *M*[*SD*] = 2.06 [0.91] “1 time per day” vs. high HA *M* [*SD*] = 3.39 [1.12] “2-4 times per day”, *t*(718) = -10.11, *P* < .01, *d* = 1.21 |  |  |  |  |
| tePoel et al., 2016 [81] | *n* = 751  clinical subsample (DSHAI ≥18) | DSHAI | frequency | Wave 1: *r* = .19, *P* < .001  Wave 2: *r* = .21, *P* < .001  Wave 3: *r* = .16, *P* < .001  Wave 4: *r* = .15, *P* < .001 | Nether-lands | not reported | no | no |
|  | *n* = 4,564  non-clinical subsample (DSHAI <18) |  |  | Wave 1: *r* = .12, *P* < .01  Wave 2: *r* = .14, *P* < .01  Wave 3: *r* = .11, *P* < .01  Wave 4: *r* = .17, *P* < .001 |  |  |  |  |
| Eichenberg & Schott, 2019 [36] | *N* = 471  general population  *n* = 190 high HA  *n* = 281 low HA  no exact cut-off reported | IAS | frequency (“more than 10 times / last year) | low HA (36.3 %) vs. high HA (76%) und da fehlen dann die konreten Werte  acute symptoms: low HA vs. high HA  (χ² = 28.82, *P* < .001)  chronic symptoms: low HA vs. high HA  (χ² = 28.17, *P* < .001) | Austria | 40.0 (13.25) | not explicity, but recruitment via online health forums | no |
