# Supplementary material for: Conceptualizations of Cyberchondria and Relations to the Anxiety Spectrum: Systematic Review and Meta-analysis
Source: J Med Internet Res. 2021 Nov 18;23(11):e27835. doi: 10.2196/27835 (PMC8663695; doi:10.2196/27835)
Supplement: Multimedia Appendix 8 [file jmir_v23i11e27835_app8.docx]

**Table A.8. Correlations between obsessive-compulsive symptoms and the Cyberchondria Severity Scale subscales.** DOCS = Dimensional Obsessive-Compulsive Scale. CSS = Cyberchondria Severity Scale. a = Norr, Oglesby et al. (2015) [18]. b = Fergus & Russell (2016) [19]. c = Bajcar et al. (2019) [40]. ** = *P* < .01 (two-tailed). * = *P* < .05 (two-tailed).

|  | | CSS | | | | | | | | | | | | | |
| --- | --- | --- | --- | --- | --- | --- | --- | --- | --- | --- | --- | --- | --- | --- | --- |
|  |  | Compulsion | | | Distress | | | Excessiveness | | | | Reassurance | | | |
|  |  | a | b | c | a | b | c | a | b | c | a | | b | c |  |
| DOCS | Contamination | .43** | .32** | .18** | .45** | .43** | .22** | .34** | .31** | .18** | | .33** | .22** | .17** |  |
|  | Responsibility | .50** | .36** | .40** | .55** | .48** | .48** | .41** | .34** | .36** | | .36** | .18** | .29** |  |
|  | Thoughts | .38** | .28** | .27** | .41** | .35** | .32** | .33** | .26** | .27** | | .28** | .08 | .14** |  |
|  | Symmetry | .36** | .27** | .27** | .40** | .27** | .34** | .29** | .15** | .23** | | .34** | .14* | .17** |  |
