# Supplementary material for: Conceptualizations of Cyberchondria and Relations to the Anxiety Spectrum: Systematic Review and Meta-analysis
Source: J Med Internet Res. 2021 Nov 18;23(11):e27835. doi: 10.2196/27835 (PMC8663695; doi:10.2196/27835)
Supplement: Multimedia Appendix 9 [file jmir_v23i11e27835_app9.docx]

**Table A.9. Emotional effects of HIU.** HA = health anxiety. HIU = health-related Internet use. SHAI = Short Health Anxiety Inventory. WI = Whiteley Index.

| **Questionnaire surveys** | | | | | | |
| --- | --- | --- | --- | --- | --- | --- |
| **First author, year** | **Sample** | **Operationalization** | | | **Type of hypothesis and analysis** | **Results** |
|  |  | **HA** | **HIU** | **Emotional effects of HIU** |  |  |
| Baumgartner & Hartmann, 2011 [13] | *N* = 104  mostly students | WI | - frequency of medical internet searches  - frequency of posting health-related information | “How did you feel after your last online health information search?”  overwhelmed, confused, frustrated, frightened, relieved, reassured | correlational hypothesis:  logistic regression | **HA as a predictor for emotional effects of HIU**  - frightened: OR = 12.25, 95% CI = 3.63-43.23, *P < .01*  - overwhelmed: OR = 6.24, 95% CI = 1.91-20.38, *P < .01*  - confused: OR = 4.04, 95% CI = 1.49-10.93, *P < .01*  - frustrated: OR = 3.13, 95% CI = 1.09-9.02, *P < .01*  - no significant relations to “relieved” or “reassured”  **Frequency as a predictor for HA post-search**  - frequency of searches: *b* = 1.17, β = 0.46, *t*(96) = 5.05, *P* < .01  - frequency of posting: *b* = 0.42, β = 0.45, *t*(96) = 4.92, *P* < .01 |
| Muse et al., 2012 [4] | *N* = 167  *n* = 46 high HA  *n* = 36 low HA  mostly students | SHAI | - frequency of HIU  - duration  - type and source of information | - post-search distress  - impact of HIU on HA (reduced/increased) | correlational hypothesis:  linear regression | **HA as a predictor for emotional effects of HIU**  - distress: β = 0.12, *P* < .001  - exacerbation of HA: β = 0.10, *P* < .05 |
|  |  |  |  |  | difference hypothesis:  group differences via  *t*-tests | **Association between HA and emotional effects of HIU**  - Distress:  low HA *M*[*SD*] = 1.75 [1.61] vs. high HA *M* [*SD*] = 4.09 [2.26],  *t*(80) = 5.25, *P* < .001  -Exacerbation of HA  low HA *M*[*SD*] = −0.92 [3.67] vs. high HA *M*[*SD*] = 1.16 [4.49],  *t*(80) = -2.23, *P* < .05 |
| Fergus, 2013 [20] | *N* = 454  general population | SHAI | - frequency of HIU | - impact of HIU on HA (reduced/increased) | correlational hypothesis:  correlation | **Association between HA and emotional effects of HIU**  *r* = .42, *P* < .01  **Association between frequency of HIU and effects of HIU**  *r* = .24, *P* < .01 |
|  |  |  |  |  | correlational hypothesis:  regression | **Frequency as a predictor for HA post-search**  *b* = 0.17, partial *r* = 0.15, *P* < .001 |
| Singh & Brown, 2014 [11] | *N* = 255  students | SHAI | - frequency of HIU  - proportion of health-related information sought  - type and source of information | - post-search tension  - post-search relief | correlational hypothesis:  correlation | **Association between HA and emotional effects of HIU**  tension: *r* = .364, *P* < .01  relief: *r* = .174, *P* < .01 |
| Doherty-Torstrick et al., 2016 [82] | *N* = 720  *n* = 640 high HA  *n* = 80 low HA  general population | WI | - duration of checking (Most  Time/ Day in the Past Month) | - increased anxiety during and post-search | correlational hypothesis: regression with  *continuous* analysis of HA | **HA as a predictor for emotional effects of HIU**  anxiety during search: β = .34, *P* < .05  anxiety after search β = .41, *P* < .05  **Duration as a predictor for emotional effects of HIU**  anxiety during search: β = .03, n.s.  anxiety after search: β = .10, *P* < .05 |
|  |  |  |  |  | correlational hypothesis:  regression with  *dichotomous* analysis of HA | **HA as a predictor for emotional effects of HIU**  anxiety during search: β = .19, *P* < .05  anxiety after search: β = .26, *P* < .05  **Duration as a predictor for emotional effects of HIU**  anxiety during search: β = .11, *P* < .05  anxiety after search: β = .18, *P* < .05 |
|  |  |  |  |  | difference hypothesis:  group differences via  *t*-tests  *continuous* analysis of HA | **Association between HA and emotional effects of HIU**   - anxiety during search: low HA *M*[*SD*] = 5.01 [1.67]   vs. high HA *M*[*SD*] = 3.78 [1.64], *t* = -6.12, *P* < .05, *d* = 0.74   - anxiety after search: low HA *M*[*SD*] = 5.02 [1.64]   vs. high HA *M*[*SD*] = 3.49 [1.61], *t* = -7.80, *P* < .05, *d* = 0.96 |
|  |  |  |  |  | difference hypothesis:  group differences via  *Χ*²-tests  *dichotomous* analysis of HA | **Association between HA and emotional effects of HIU**   - worsened anxiety during search: 68.3% of high HA   vs. 40.0% of low HA, χ² = 25.05, *P* < .01   - worsened anxiety after search: 67.2% of high HA   vs. 28.8% of low HA, χ² = 45.03, *P* < .01 |
|  |  |  |  |  | difference hypothesis: group difference  *dichotomous* analysis of duration (long vs. short) | - heightened anxiety during search: 51.9% of short duration   vs. 72.1% of long duration, *t* = 17.9, *P* < .01   - heightened anxiety after search: 46.0% of short duration   vs. 71.7% of long duration, *t* = 28.1, *P* < .01 |
| Eichenberg & Schott, 2019 [36] | *N* = 471  general population  *n* = 190 high HA  *n* = 281 low HA  no exact cut-off reported | WI | - type of online health service | - potential of different online health services to alleviate HA | difference hypothesis:  group difference via *t*-test  *dichotomous* analysis of HA | **Source as a predictor for emotional outcome**   - individuals with high HA did not rate the impact of online health services significantly different to individuals with low HA (*P*s = .04 - .88)   **Whole sample**   - sharing with other persons affected (*M* = 1.94, *SD* = .83) and online contact with experts (*M* = 1.93, *SD* = .83) were able to alleviate anxiety - online diagnosis systems (*M* = 1.10, *SD* = .77) and video platforms (*M* = 0.98, *SD* = .83) seemed to cause increased anxiety |
